# Biomimetic Hydrogel System Targeting S100A8 Centered Neuroimmune Crosstalk and Hypoxia Induced Neuronal Injury

**DOI:** 10.1002/advs.76220

**Published:** 2026-06-22

**Authors:** Peng Liu, Xiaoyang Wu, Xiaoyin Liu, Yuyan Wang, Shichao Jiang, Kai Wu, Gaowei Li, Jie Ding, Chengheng Wu, Dan Wei, Jing Sun, Hongsong Fan, Liangxue Zhou

**Affiliations:** ^1^ Department of Neurosurgery West China Medical School West China Hospital Sichuan University Chengdu Sichuan China; ^2^ National Engineering Research Center for Biomaterials College of Biomedical Engineering Sichuan University Chengdu Sichuan China; ^3^ Department of Neurosurgery NHC Key Laboratory of Nuclear Technology Medical Transformation (Mianyang Central Hospital) School of Medicine University of Electronic Science and Technology of China Mianyang Sichuan China

**Keywords:** biomimetic hydrogel, neuroimmune crosstalk, S100A8, TBI repair

## Abstract

Tissue hypoxia and neuroinflammation are major drivers of secondary injury after traumatic brain injury (TBI). S100A8, a pro‐inflammatory damage‐associated molecular pattern, is involved in pathological neuron–microglia signaling and may amplify secondary damage. However, effective interventions targeting S100A8‐centered neuroimmune crosstalk remain lacking. Here, we developed a multifunctional biomimetic hydrogel system, HPC@Gel, by integrating modified hemoglobin nanoparticles and curcumin‐based carbon quantum dots into a hyaluronic acid–collagen hydrogel to disrupt this vicious cycle. In vitro, hypoxic HT22 neurons, LPS‐stimulated BV2 microglia, and an HT22–BV2 Transwell model were used to evaluate neuroprotection and neuroimmune regulation. HPC@Gel markedly downregulated S100A8 expression and attenuated pathological neuron–microglia crosstalk. Recombinant S100A8 rescue experiments further showed that exogenous S100A8 partially reversed the protective effects of HPC@Gel, supporting the functional role of S100A8 suppression. In a rat cortical cavity TBI model, HPC@Gel reduced S100A8 expression in neurons and microglia, alleviated neuronal hypoxia, and reversed neuroinflammation. These effects improved the pathological microenvironment, promoted endogenous neural regeneration, and facilitated neurological and cognitive recovery. This study provides a promising therapeutic strategy for TBI by simultaneously targeting hypoxia and S100A8‐mediated neuroimmune crosstalk.

## Graphical abstract

A biomimetic hydrogel (HPC@Gel) was developed for traumatic brain injury repair by combining oxygen‐carrying hemoglobin nanoparticles and anti‐inflammatory carbon quantum dots within a brain‐like matrix. The system alleviated hypoxia, reduced harmful neuroimmune signaling, protected neurons, supported neural regeneration, and improved neurological and cognitive outcomes in vivo.

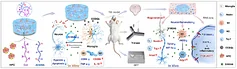

## 1 Introduction

Traumatic brain injury (TBI) pathology consists of the initial primary insult and a subsequent secondary injury cascade that unfolds over hours to days, critically influencing long‐term outcomes [1, 2, 3]. Secondary injury has therefore become the principal target for therapeutic interventions [4, 5]. Generally, this phase is characterized by cerebral hypoxia and neuroinflammation, which collectively disrupt the homeostatic balance between neurons and microglia [6]. These pathological interactions are regulated by damage‐associated molecular patterns (DAMPs). Among them, S100A8 is a potent DAMP that serves as a key mediator of pathological neuronal–microglial crosstalk and is closely associated with poor prognosis in TBI. However, current research has not yet identified effective interventions to target the S100A8‐centered neuroimmune crosstalk pathway.

Mechanistically, markers of secondary injury, such as hypoxia, trigger the expression and release of S100A8 in damaged neurons [7, 8]. Once released, extracellular S100A8 activates microglia through TLR4 and RAGE signaling, thereby amplifying pro‐inflammatory cytokine production and exacerbating neuronal loss [9, 10]. This process establishes a deleterious feed‐forward loop that directly links hypoxia to microglial overactivation. Consequently, an ideal therapeutic strategy should not only alleviate neuroinflammation and hypoxia but also disrupt the S100A8‐mediated signaling axis to halt the secondary injury cascade and enhance neurological recovery. Based on this rationale, our design does not rely on suppressing S100A8 alone; instead, it aims to simultaneously improve the upstream hypoxic microenvironment and attenuate downstream inflammatory amplification.

Curcumin‐derived carbon quantum dots (CCQDs), with their improved water solubility, biocompatibility, and potent anti‐inflammatory properties, represent an attractive agent for attenuating neuroinflammation [11, 12, 13]. Recent studies have demonstrated that curcumin and its derivatives can target the S100A8 protein and reduce its expression in arthritis and chronic myeloid leukemia, thereby modulating both local and systemic inflammatory responses. Accordingly, it is reasonable to hypothesize that CCQDs may exert a similar inhibitory effect on S100A8 expression in TBI [14, 15]. However, it is still unclear whether CCQDs can block the neuroimmune crosstalk centered on S100A8 in TBI, which makes it a worthy and highly promising therapeutic target for research. Since hypoxia is a primary driver of S100A8 expression in TBI, alleviating the hypoxic microenvironment can also downregulate this pathway. To achieve this, the introduction of oxygen‐carrying biomaterials has emerged as a promising therapeutic strategy. Hemoglobin (HB), owing to its intrinsic physiological oxygen delivery capacity, has attracted considerable attention. However, free HB is prone to autoxidation, resulting in the formation of methemoglobin, which lacks oxygen transport ability and simultaneously induces oxidative stress [16]. Catechol‐mediated surface modification not only stabilizes the oxygen‐binding capability of HB but also provides a versatile platform for conjugation with functional nanomaterials such as CCQDs. This design enables the development of a bifunctional nanoplatform capable of both inflammation suppression and oxygen release. Nonetheless, efficient and sustained delivery of such therapeutic agents to the lesion site remains a major challenge due to the complex brain microenvironment and biological barriers [17].

Interestingly, the parenchymal cavity formed after TBI offers a natural niche for localized therapeutic delivery. Hydrogel‐based biomaterials have garnered particular interest in this regard, owing to their tissue‐mimetic composition, tunable mechanical properties, and ability to fill irregular defects [18, 19]. Hydrogels designed to recapitulate brain extracellular matrix (ECM) composition, stiffness, and viscoelasticity can serve as biomimetic scaffolds that not only promote tissue integration but also provide a sustained release platform for bioactive agents [20]. Incorporating oxygen‐releasing and anti‐inflammatory nanocomponents into such ECM‐mimicking hydrogels offers a unique opportunity to remodel the pathological microenvironment in TBI and interrupt secondary injury cascades.

**SCHEME 1:**
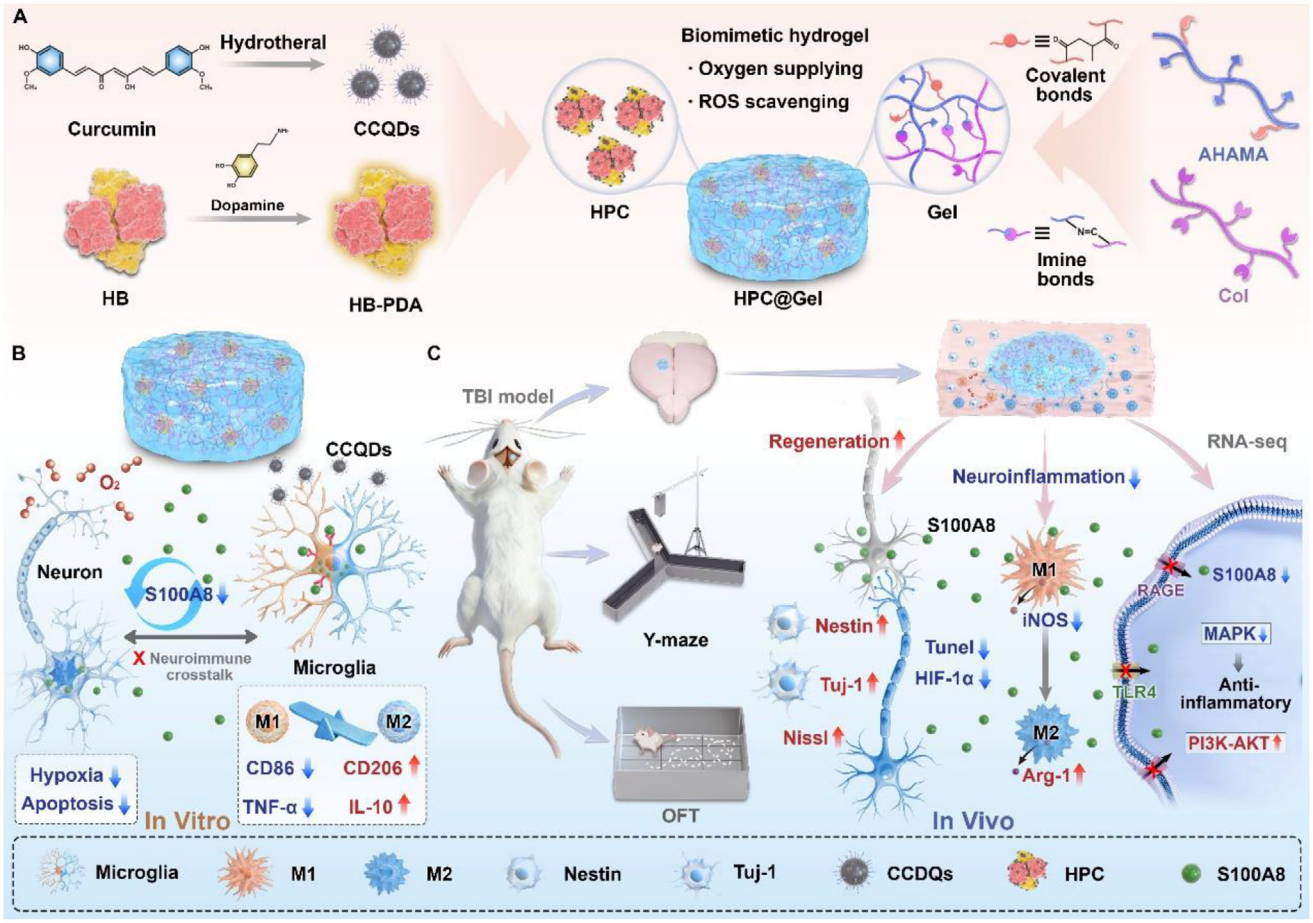
(A) Synthesis of the biomimetic dual‐network hydrogel system loaded with HPC. (B) In vitro verification that HPC@Gel effectively interrupts the S100A8‐centered neuroimmune crosstalk, protects neurons, and modulates neuroinflammatory responses. (C) In vivo assessment of the biomimetic gel system in a TBI animal model, showing improved neurological function and restoration of the local injury microenvironment.

Herein, we report the design and fabrication of a multifunctional, biomimetic hydrogel system (HPC@Gel) engineered to simultaneously mitigate tissue hypoxia and disrupt detrimental S100A8‐mediated neuroimmune crosstalk in TBI (Scheme 1). Specifically, HB is conjugated to CCQDs via polydopamine‐mediated surface modification, resulting in a dual‐functional nanoconjugate with both oxygen‐releasing and inflammation‐attenuating capabilities. The resulting nanoconjugate (HPC), synthesized through a one‐step reaction, could be readily integrated into a biomimetic hydrogel of dual‐functionalized hyaluronic acid (aldehyde‐ and methacrylate‐modified) / collagen (Gel). This biomimetic system effectively alleviates tissue hypoxia while simultaneously disrupting detrimental S100A8‐mediated neuroimmune crosstalk following TBI. We demonstrated that this system effectively protected neurons from hypoxic apoptosis and modulated microglial polarization toward a pro‐reparative M2 phenotype by downregulating S100A8 expression. Crucially, implantation of the HPC@Gel into a rat TBI model significantly attenuated local neuroinflammation, enhanced endogenous neural stem cell proliferation and differentiation, and ultimately led to substantial recovery of neurological and cognitive functions. This work thus presents a promising therapeutic strategy, demonstrating that comprehensively modulating the complex post‐injury microenvironment through an engineered biomaterial can effectively promote neural regeneration and functional repair in TBI.

## 2 Results and Discussion

### 2.1 Preparation and Characterization of a Biomimetic Hydrogel Scaffold With Synergistic Antioxidant and Oxygen‐releasing Functions

**FIGURE 1:**
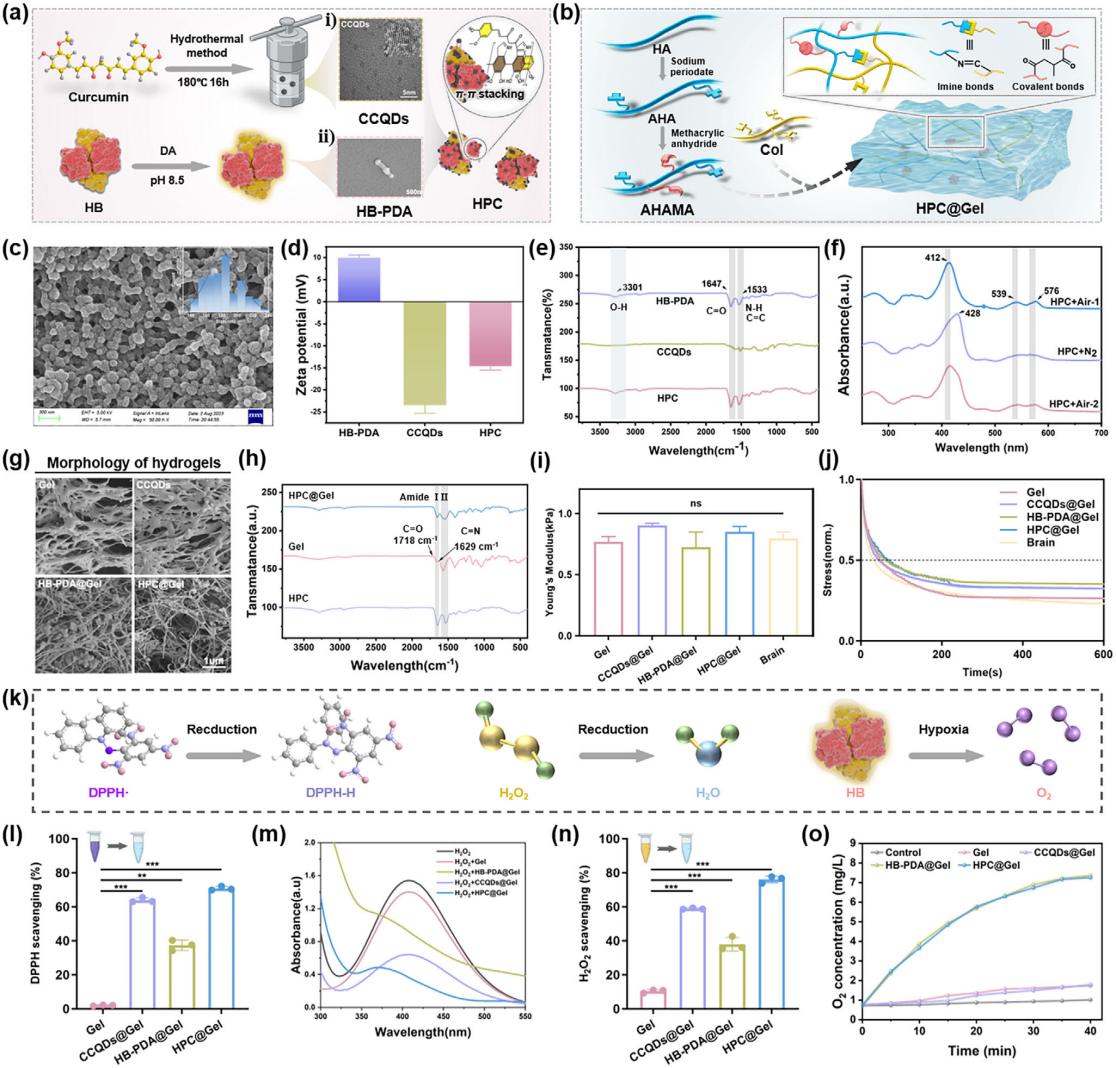
Preparation and structural–functional characterization of HPC@Gel. (a) Schematic illustration of the preparation of HPC: (i) TEM image of CCQDs, (ii) SEM image of HB‐PDA. (b) Schematic illustration of the preparation of Gel and HPC@Gel; (c) SEM image of HPC with particle size distribution shown in the inset; (d) Zeta potential and (e) FTIR spectra of HB‐PDA, CCQDs, and HPC; (f) UV–vis spectra of HPC showing the characteristic Soret band (412 nm) and Q bands (539 and 576 nm). The reversible shift of the Soret band during deoxygenation and re‐oxygenation indicates retained oxygen‐binding responsiveness of HPC; (g) SEM images and (h) FTIR spectra of HPC, Gel, and HPC@Gel. The imine bonding (C═N) at 1629 cm^−^^1^ in the Gel group confirms the interactions between Col and HA. The C═O shoulder peak (1718 cm^−^^1^) derived from methacrylate was masked by the broad PDA signal, whereas the amide I (1660 cm^−^^1^) and amide II (1569 cm^−^^1^) bands of collagen overlapped with those of HB and exhibited redshifts (to 1654 cm^−^^1^) and broadening. (i) statistical analysis of Young's modulus, and (j) stress‐relaxation curves of composite hydrogels; (k) Schematic representation of the antioxidant and oxygen‐release functions of HPC@Gel; (l) DPPH scavenging efficiencies. (m) UV–vis spectra and (n) statistical results showing H_2_O_2_ scavenging activity of the composite hydrogels. (o) Oxygen release profiles of the composite hydrogels. Data are presented as mean ± SD from n = 3 independent experiments. Statistical significance was analyzed using one‐way ANOVA followed by Tukey's multiple‐comparison test. ns, no significant difference; ^*^ *p* < 0.05; ^**^ *p* < 0.01; ^***^ *p* < 0.001; ^****^ *p* < 0.0001.

To prepare the composite hydrogel scaffold, we first synthesized composite nanoparticles of CCQDs and HB (HPC) and the dual‐network biomimetic hydrogel (Figure 1a,b). Curcumin carbon quantum dots (CCQDs) were synthesized by hydrothermal treatment of curcumin aqueous solution (40 mg/mL) at 180°C for 12 h, followed by centrifugation, 0.22 µm filtration, dialysis (MWCO 1 kDa), and lyophilization. TEM showed a lattice spacing of 0.21 nm corresponding to the (100) plane of graphitic carbon [21]. Compared with native curcumin, which is limited by poor aqueous dispersibility, CCQDs exhibited markedly improved water dispersibility, facilitating their homogeneous incorporation into the hydrogel system (Figure S1). Their radical‐scavenging activity was evaluated separately in the functional characterization experiments. HPC was fabricated by incorporating 100 µg/mL CCQDs during the dopamine oxidative polymerization on the HB surface. HPC exhibited a uniform size distribution (185.3 ± 25.8 nm, Figure 1c). The variation of surface charge of HB‐PDA and HPC evidenced the successful integration of CCQDs in HPC (Figure 1d). To further assess the colloidal stability of HPC, the hydrodynamic diameter and zeta potential of the nanoparticles were monitored in PBS over 7 days (days 0, 3, 5, and 7). No significant changes were observed in either parameter during this period, indicating that HPC remained colloidally stable under physiological buffer conditions (Figure S2). A suitable cytocompatible concentration of CCQDs for subsequent nanoparticle preparation and in vitro evaluation was determined by the CCK‐8 assay (Figure S3). Besides, the Fourier transform infrared (FTIR) spectroscopy indicated the retention of characteristic protein‐related functional groups after nanoparticle assembly and suggested hydrogen bonding and aromatic interactions (*π–π* stacking) between CCQDs and HB‐PDA in HPC (Figure 1e) [22]. The UV–vis spectra of HPC showed characteristic hemoglobin absorption features, including the Soret band and Q bands. The reversible shift of the Soret band from 412 nm (oxygenated) to 428 nm (deoxygenated), and its recovery to 412 nm upon re‐oxygenation, supports retained oxygen‐binding responsiveness after nanoparticle assembly (Figure 1f) [23].

The hydrogel matrix (Gel) from aldehyde‐modified and methacrylated hyaluronic acid (HA) and collagen (Col) was constructed as shown in Figure 1a,b. ^1^H NMR spectra validated the successful synthesis of the functionalized HA (Figure S4). Upon incorporation of HPC, the hydrogel system underwent gelation through dynamic imine bonds and photo‐crosslinked covalent bonds (HPC@Gel). SEM imaging revealed a loose and porous fibrous network architecture with uniformly distributed nanoparticles, favorable for cellular seeding and functional interactions (Figure 1g). FTIR analysis supported the hypothesis that the gel is crosslinked by covalent imine bonds, whereas the HPC composite system achieves its integrated structure through a network of hydrogen bonds (Figure 1h) [24]. Mechanical testing demonstrated that the composite hydrogels with varied components exhibited similar Young's moduli (∼1 kPa, Figure 1i) and stress relaxation behaviors (Figure 1j). Specifically, Young's modulus was obtained from unconfined compression testing using a dynamic mechanical analyzer (DMA), whereas stress relaxation was measured on a rheometer under 10% compressive strain for 600 s. These mechanical parameters fall within the physiological range of natural brain tissue, thereby providing a mechanically compatible microenvironment for nerve cells. Given the highly compliant and viscoelastic nature of brain tissue, such time‐dependent mechanical behavior is relevant because it may facilitate cell adaptation, reduce local mechanical mismatch, and support tissue integration after implantation [25, 26]. Although the incorporation of CCQDs, HB‐PDA, or HPC introduced additional local interactions, their loading level was not sufficient to substantially alter the bulk crosslink density of the HA/Col network; thus, the overall modulus and relaxation behavior remained comparable across groups. In addition, comparable degradation rates and swelling profiles were observed across groups. When incubated at 37°C, the hydrogels showed a similar degradation degree in type I collagenase solution after 72 h (74.8 ± 2.3%) and a comparable swelling ratio in PBS at 72 h (14.7 ± 1.2%) (Figure S5). The moderate degradation enables gradual breakdown aligned with tissue healing, while the controlled swelling ensures mechanical compatibility with soft brain tissue, reducing pressure risks and enhancing neural integration.

HPLC analysis of CCQDs release from the HPC@Gel system revealed a cumulative release rate of 77.4 ± 2.2% within 72 h. This early sustained‐release profile is biologically relevant because it overlaps with the acute phase of secondary injury after TBI, during which hypoxia, inflammatory amplification, and neuronal apoptosis are particularly active. This rate was slightly lower than that observed for the hydrogel containing only CCQDs (89.3 ± 0.6%), which may be attributed to the non‐covalent interactions between CCQDs and the HB‐PDA nanoparticles, leading to a more sustained release compared to the simple diffusion of free CCQDs from the hydrogel matrix (Figure S6). Given the bifunctionality of the HPC@Gel, the antioxidant and oxygen‐release properties of the composite hydrogel scaffold were systematically characterized (Figure 1k). HPC@Gel exhibited remarkable scavenging activity toward both reactive oxygen species (ROS) and reactive nitrogen species (RNS) [27, 28]. In both H_2_O_2_‐ and DPPH‐scavenging assays, the synergistic effects of HB‐PDA and CCQDs resulted in efficient radical elimination (Figure 1l–n and Figure S7). The oxygen release profiles indicated that under hypoxic conditions, the oxygen concentrations of HPC@Gel and HB‐PDA@Gel showed no significant difference, with a maximum value of 7.36 mg/L, indicating that oxygen release was mainly derived from the HB‐containing component (Figure 1o). Although CCQDs did not further increase oxygen output, they contributed additional antioxidant activity. Therefore, the bifunctionality of HPC@Gel is reflected in the integration of oxygen‐release and radical‐scavenging properties rather than in synergistic enhancement of oxygen release itself. In summary, the successful integration of bifunctional nanoparticles HPC endowed the bioactive biomimetic hydrogel scaffolds with the potential to ameliorate microenvironmental neuroinflammation and hypoxia.

### 2.2 The Biomimetic Gel System Alleviates Neuronal Hypoxia, Inhibits Neuroinflammation, and Enhances In Vitro Neuroprotection

Hypoxia in brain tissue following TBI is a key pathological factor that triggers neuronal apoptosis and irreversible secondary damage [29]. Thus, improving neuronal oxygen supply and suppressing hypoxia‐induced apoptosis are critical therapeutic goals for mitigating secondary injury and improving outcomes in TBI patients. To evaluate whether the biomimetic hydrogel system exerts direct protective effects under hypoxic conditions, we employed the mouse hippocampal neuronal cell line HT22, cultured in a hypoxic microenvironment to simulate post‐TBI conditions. Before mechanistic evaluation, CCK‐8 assays were used only to determine a suitable CCQD concentration and to assess the baseline cytocompatibility of the hydrogel formulations. The results demonstrated no significant differences in cell viability among the scaffold groups on days 1, 3, and 5, indicating acceptable cytocompatibility of the biomimetic gel (Figure S3b).

To directly assess intracellular oxygenation, cells were further stained with the oxygen‐sensitive probe Ru(dpp)_3_(PF_6_)_2_ (Figure S8). No significant difference in fluorescence intensity was observed among the Hypoxia, Gel, and CCQDs@Gel groups, whereas both HB‐PDA@Gel and HPC@Gel showed significantly reduced fluorescence intensity. These findings indicate that the improvement in intracellular oxygenation was mainly attributable to the hemoglobin‐containing component rather than CCQDs alone.

**FIGURE 2:**
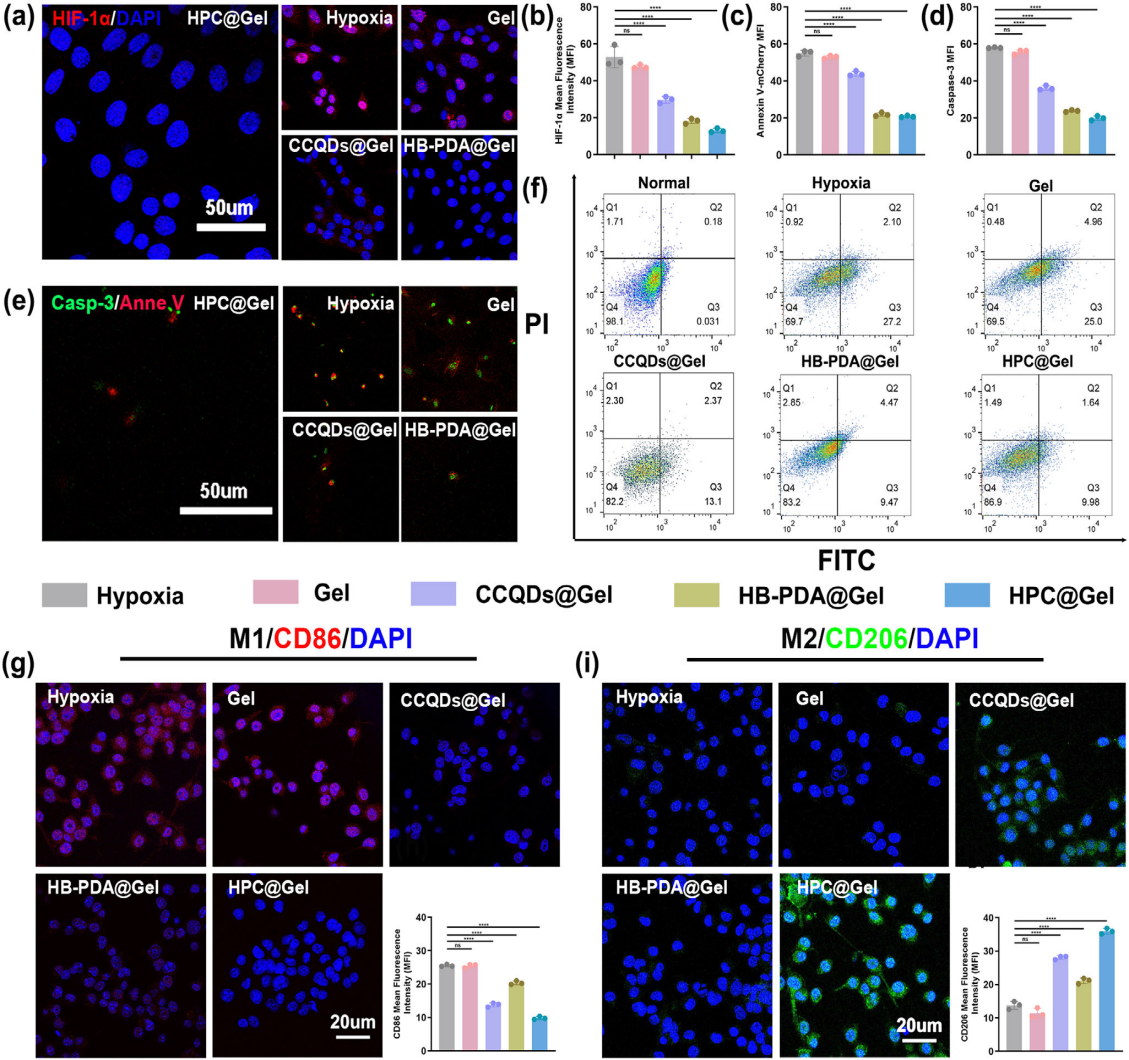
The bionic composite hydrogel ameliorates hypoxia, reduces neuronal apoptosis, and mitigates neuroinflammation in vitro. (a) Representative immunofluorescence images of HIF‐1α (red), DAPI (blue) in hypoxic HT22 cells after 24 h of co‐culture with different hydrogel formulations. (b) Quantitative analysis of the MFI of HIF‐1α from images in (a). (c,d) Quantitative analysis of the MFI of Annexin V and Caspase‐3 from images in (e). (e) Representative immunofluorescence images co‐staining for Annexin V (red) and cleaved Caspase‐3 (green) in hypoxic HT22 cells. Co‐localization indicates active apoptosis. (f) Flow cytometry analysis of HT22 apoptosis using Annexin V‐FITC/PI staining, “Normal” refers to cells cultured under normoxic conditions. BV2 microglia were stained for the (g) M1 marker CD86 (red) or the (i) M2 marker CD206 (green), and DAPI (blue). Quantitative analysis of the mean fluorescence intensity (MFI) of (h) CD86 and (j) CD206 from images in (g,i), respectively. Data are presented as mean ± SD from n = 3 independent experiments. Statistical significance was analyzed using one‐way ANOVA followed by Tukey's multiple‐comparison test. ns, no significant difference; ^*^ *p* < 0.05; ^**^ *p* < 0.01; ^***^ *p* < 0.001; ^****^p < 0.0001.

HIF‐1α is the central transcription factor mediating cellular responses to hypoxia, and it accumulates under oxygen deprivation. As shown in Figure 2a,b, both the hypoxia control group and the blank hydrogel (Gel) group exhibited markedly elevated HIF‐1α expression, confirming successful hypoxia model establishment. By contrast, HIF‐1α levels were significantly reduced in the HB‐PDA@Gel and HPC@Gel groups. Together with the intracellular oxygen‐probe results, these findings support alleviation of cellular hypoxia by the HB‐containing formulations. Collectively, these results suggest that the HB‐containing formulations improved intracellular oxygenation, whereas CCQDs may additionally modulate hypoxia signaling pathways [30].

We next investigated whether the alleviation of hypoxic stress could enhance neuronal survival. Apoptosis, a major form of programmed cell death, depends on proteases such as Caspase‐3 and is characterized in early stages by phosphatidylserine (PS) externalization. As shown in Figure 2c–e, by GreenNuc‐labeled Caspase‐3 and mCherry‐labeled Annexin V to identify apoptotic cells, we can observe the hypoxia induced widespread apoptosis in HT22 cells, as indicated by strong Caspase‐3 and Annexin V signals [31]. In contrast, all nanoparticle‐loaded hydrogel systems attenuated apoptosis to varying degrees, with HPC@Gel providing the strongest protective effect. This trend paralleled the changes in HIF‐1α and intracellular oxygenation, consistent with reduced hypoxic stress as a contributor to its anti‐apoptotic effect. Flow cytometry with Annexin V‐FITC/PI staining, with the normoxic group (Normal) as a baseline control, further confirmed that hypoxia markedly increased HT22 apoptosis, whereas hydrogel treatment attenuated this effect, with HPC@Gel showing the strongest protection (Figure 2f).

During the acute phase of TBI, microglia act as key immune sentinels but undergo phenotypic imbalance under pathological stress, shifting toward pro‐inflammatory M1 polarization. M1 microglia release high levels of cytokines such as tumor necrosis factor‐α (TNF‐α), thereby amplifying neuroinflammation and exacerbating secondary brain damage [32]. Accordingly, regulating microglial polarization to restore the M1–M2 balance is a promising therapeutic strategy. To test whether HPC@Gel modulates microglial activation, we used the BV2 microglial cell line to induce M1 polarization with lipopolysaccharide (LPS). As shown in immunofluorescence results (Figure 2g,i), LPS‐treated BV2 cells exhibited increased CD86 and decreased CD206 expression, validating the in vitro neuroinflammation model. Treatment with CCQDs@Gel or HPC@Gel reversed this phenotype: CD86 was downregulated and CD206 upregulated, with HPC@Gel showing the strongest effect (Figure 2h,j). This enhanced efficacy derives not from additive drug action but from the rationally designed synergy of multiple bioactive components. Specifically, CCQDs demonstrated notable anti‐inflammatory activity, which was further enhanced when combined with HB‐PDA nanoparticles [22, 33]. Thus, the superior performance of HPC@Gel reflects its multifunctional composition, promoting a more effective shift of microglia from the M1 phenotype to the M2 phenotype. ELISA further confirmed reduced TNF‐α secretion and increased IL‐10 production in BV2 cells after treatment (Figure S9). Together, these results demonstrate that HPC@Gel effectively reverses LPS‐induced M1 polarization, suppresses pro‐inflammatory activity, and promotes M2‐like anti‐inflammatory transformation, providing a cellular basis for attenuating neuroinflammation after TBI [34].

In summary, the biomimetic hydrogel system exerted dual neuroprotective effects by promoting microglial polarization to reduce neuroinflammation and by attenuating hypoxia‐associated stress and apoptosis. This dual mechanism targets two major drivers of secondary TBI injury, highlighting the potential of the system for enhanced therapeutic efficacy in complex in vivo conditions. Collectively, the in vitro findings provide robust evidence supporting the capacity of the biomimetic hydrogel system to improve the post‐TBI pathological microenvironment.

### 2.3 Neuronal–Microglial Crosstalk Mediated by S100A8 and its Blockade by the Biomimetic Gel System

**FIGURE 3:**
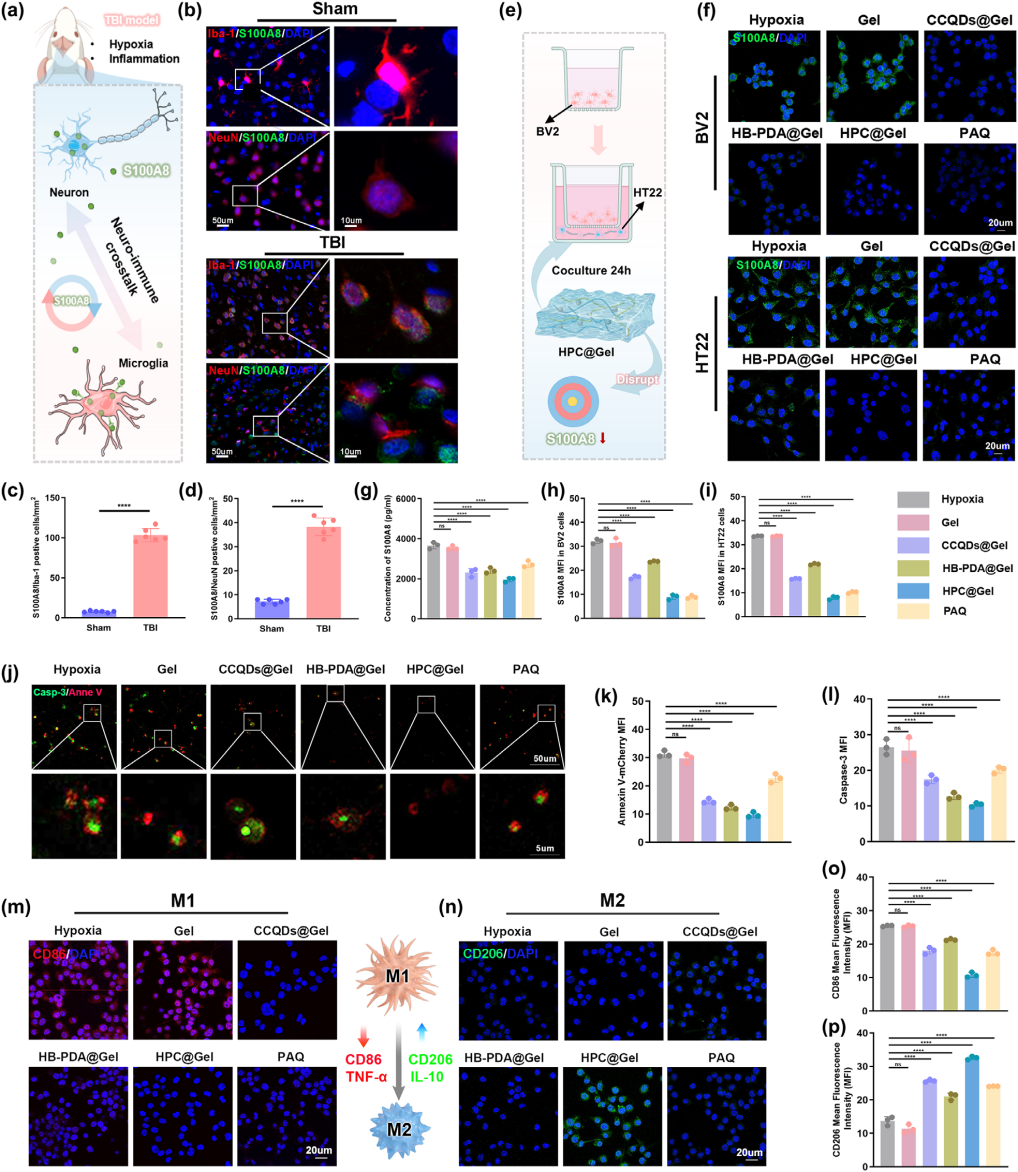
Biomimetic hydrogel blocks S100A8‐mediated microglia‐neuron crosstalk, alleviating neuroinflammation and neuronal apoptosis. (a) Schematic illustration of S100A8‐mediated neuroimmune crosstalk in TBI. (b) Representative immunofluorescence images showing co‐localization of S100A8 (green) with the Iba‐1 (red) and the NeuN (red) in brain sections collected from the injured cortical region of rats subjected to the cortical cavity TBI model, with sham‐operated rats as controls. Nuclei were counterstained using DAPI, which appears blue in the images. (c,d) Quantitative analysis of the number of S100A8+/Iba‐1+ cells and S100A8+/NeuN+ cells in the brain sections shown in (b). (e) Schematic illustration of HPC@Gel blocks crosstalk during co‐culture of HT22 and BV2 cells. (f) Representative immunofluorescence images of S100A8 (green) in BV2 and HT22 cells subjected to hypoxia and treated with different formulations. (g) ELISA quantification of secreted S100A8 levels in the culture medium from the experiment in (f). Quantitative analysis of the MFI of S100A8 in (h) BV2 cells and (i) HT22 cells from images in (f). (j) Representative immunofluorescence images of apoptosis in HT22 cells, co‐stained for Annexin V (red) and cleaved Caspase‐3 (green). Quantitative analysis of the MFI of (k) Annexin V and (l) Caspase‐3 from images in (j), representing the Transwell co‐culture model of HT22 and BV2 cells. (m,n) Representative immunofluorescence images of microglia polarization in BV2 cells. CD86 (red), CD206 (green), and nuclei with DAPI (blue). Quantitative analysis of the MFI of (o) CD86 and (p) CD206. Data are presented as mean ± SD from n = 3 independent experiments. Statistical significance was analyzed using one‐way ANOVA followed by Tukey's multiple‐comparison test. ns, no significant difference; ^*^ *p* < 0.05; ^**^ *p* < 0.01; ^***^ *p* < 0.001; ^****^ *p* < 0.0001.

During the pathological progression of TBI, neurons and microglia do not function independently but engage in maladaptive signal crosstalk, forming a vicious cycle that exacerbates injury. The S100A8 protein, an endogenous DAMP, appears to participate in this interaction. Under hypoxic conditions, neurons secrete S100A8, which activates microglia and, in turn, further damages neurons (Figure 3a) [35]. This mechanism represents both a pathological driver and a potential therapeutic target. We therefore hypothesized that the composite biomimetic hydrogel system could disrupt S100A8‐centered neuroimmune crosstalk, suppress S100A8 expression, alleviate neuroinflammation, and provide neuroprotection.

To determine whether S100A8 is involved in pathological neuroimmune signaling after TBI, we first examined its expression in vivo using brain tissue collected from the injured cortical region of rats subjected to the cortical cavity TBI model, with sham‐operated animals serving as controls. As shown in Figure 3b, co‐localization of S100A8 with neurons (NeuN^+^) and microglia (Iba‐1^+^) was markedly increased in TBI brain tissue compared with the sham group, with microglia showing a 13.5‐fold increase in co‐localized cell number and neurons showing a 5.3‐fold increase (Figure 3c,d). Based on this in vivo observation, we next established in vitro models using hypoxia‐treated HT22 neurons, inflammatory BV2 microglia, and an HT22–BV2 Transwell co‐culture system to investigate whether the biomimetic hydrogel could regulate S100A8‐associated neuron–microglia crosstalk under controlled conditions. ELISA analysis further revealed that free S100A8 protein was detectable only in the supernatant of hypoxia‐treated HT22 neurons, but not BV2 microglia, indicating that neuronal secretion drives elevated S100A8 under hypoxia (Figure S10).

We then used the HT22–BV2 Transwell system together with Paquinimod (PAQ), a selective S100A8 inhibitor, to further examine whether S100A8 mediates this crosstalk (Figure 3e) [36]. In the hypoxia group, fluorescence intensity of S100A8 was strongly increased, whereas PAQ treatment (10 µm) significantly reduced its expression and secretion (Figure 3f). Similarly, HPC@Gel treatment suppressed S100A8 expression, with ELISA confirming consistent reductions in protein secretion (Figure 3g). These findings show that the biomimetic hydrogel system effectively suppresses S100A8 expression in both neurons and microglia under the present experimental conditions. Notably, the inhibitory effect exerted by CCQDs@Gel was substantially more potent than that of HB‐PDA@Gel, suggesting that CCQDs are the primary active component responsible for this specific therapeutic action (Figure 3h,i). This result not only confirms the bioactivity of CCQDs as hypothesized but also clarifies that the strong efficacy of nanoparticle HPC in reducing S100A8 is largely driven by the potent function of its CCQD component. As a tightly regulated DAMP, S100A8 expression is controlled by cellular stress and inflammatory pathways. In TBI, its upregulation activates neuroinflammation through multiple signaling cascades [37]. The observed suppression of S100A8 by HPC@Gel therefore suggests that its bioactive components inhibit upstream stress pathways at the transcriptional level, conferring both anti‐inflammatory and antioxidant effects.

The above experiments confirmed that HPC@Gel effectively suppresses the expression of the pro‐inflammatory mediator S100A8. Building on this finding, we next examined whether this molecular‐level modulation could lead to functional neuroprotective effects. As shown in Figure 3j–l, HPC@Gel treatment markedly reduced the number of apoptotic HT22 cells. Additionally, immunofluorescence and ELISA assays on BV2 cells revealed that M1‐related markers (CD86, TNF‐α) decreased, while M2‐related markers (CD206, IL‐10) increased (Figure 3m–p and Figure S11). The superior therapeutic outcome of HPC@Gel is attributable to the combined action of these distinct yet synergistic functionalities. We discovered that component HB‐PDA exhibited a more potent anti‐apoptotic effect than CCQDs, suggesting it is the primary agent for direct neuronal protection. Whereas CCQDs were significantly more effective than component HB‐PDA in modulating inflammatory response, highlighting their principal role in immunomodulation. Taken together, these functional results, along with the observed inhibition of S100A8 shown in Figure 3, provide compelling evidence that HPC@Gel exerts its therapeutic effects by blocking the harmful neuronal–microglial crosstalk mediated by S100A8.

In summary, these findings validate our hypothesis: the biomimetic hydrogel system disrupts S100A8‐centered neuronal–microglial crosstalk, mitigates neuroinflammation, and reveals a synergistic neuroprotective mechanism driven by CCQDs and HB‐PDA. The HPC@Gel system alleviates neuronal hypoxia, thereby not only inhibiting intrinsic apoptotic pathways but also potentially suppressing upstream stress signals, such as DAMPs, that trigger microglial activation. This upstream regulation, together with the direct anti‐inflammatory action of its active components on microglia, establishes a beneficial feedback loop that disrupts the vicious cycle of secondary injury.

### 2.4 Recombinant S100A8 Rescue Partially Reverses the Neuroprotective and Immunomodulatory Effects of HPC@Gel

**FIGURE 4:**
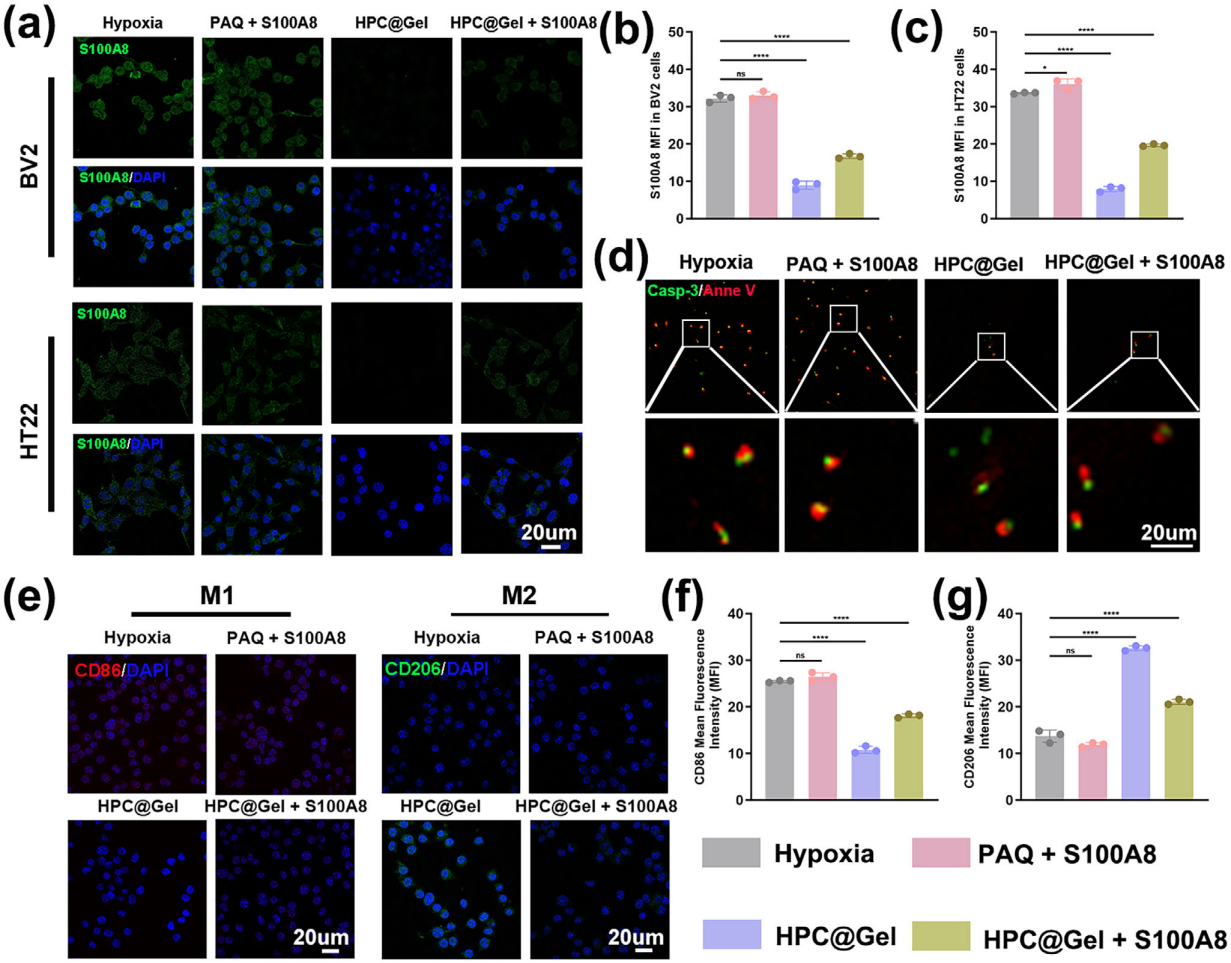
(a) Representative immunofluorescence images of S100A8 protein expression (green) in BV2 and HT22 cells after different treatments under hypoxic conditions; nuclei were counterstained with DAPI (blue). (b) Quantitative analysis of the MFI of S100A8 in BV2 cells. (c) Quantitative analysis of the MFI of S100A8 in HT22 cells. (d) Representative immunofluorescence images of apoptotic HT22 cells, co‐stained for Caspase‐3 (green) and Annexin V (red). (e) Representative immunofluorescence images of M1 and M2 markers in BV2 cells, stained for CD86 (red), CD206 (green), and DAPI (blue). (f) Quantitative analysis of CD86 MFI in BV2 cells shown in (e). (g) Quantitative analysis of CD206 MFI in BV2 cells shown in (e). Data are presented as mean ± SD from *n* = 3 independent experiments. Statistical significance was analyzed using one‐way ANOVA followed by Tukey's multiple‐comparison test. ns, no significant difference; ^*^ *p* < 0.05; ^**^ *p* < 0.01; ^***^ *p* < 0.001; ^****^ *p* < 0.0001.

To further determine whether the neuroprotective and anti‐inflammatory effects of HPC@Gel depend on suppression of S100A8, we performed a rescue experiment by exogenously adding recombinant S100A8 protein in the HT22‐BV2 co‐culture system. As shown in Figure 4, S100A8 expression was markedly increased in both BV2 and HT22 cells under hypoxic conditions, accompanied by enhanced HT22 apoptosis and a shift of BV2 cells toward a pro‐inflammatory M1 phenotype (Figure 4a–g). Compared with the Hypoxia group, HPC@Gel treatment significantly reduced the mean fluorescence intensity of S100A8, decreased HT22 apoptosis, and promoted polarization of BV2 cells toward an anti‐inflammatory M2 phenotype, as evidenced by increased CD206 fluorescence intensity. When exogenous S100A8 was added back to the HPC@Gel‐treated system (HPC@Gel + S100A8 group), S100A8 expression was partially restored, while HT22 apoptosis and CD86 expression in BV2 cells increased significantly, together with a reduction in CD206 expression. These findings indicate that exogenous S100A8 partially reversed the protective effects of HPC@Gel. A similar rescue pattern was observed in the PAQ group: supplementation with recombinant S100A8 in the presence of PAQ (PAQ + S100A8 group) restored S100A8 expression, apoptosis, and M1 markers to levels close to those of the Hypoxia group, further indicating that the protective effect of PAQ is highly dependent on suppression of S100A8.

### 2.5 Biomimetic Hydrogel System Facilitates Neural Regeneration and Functional Recovery Following TBI

**FIGURE 5:**
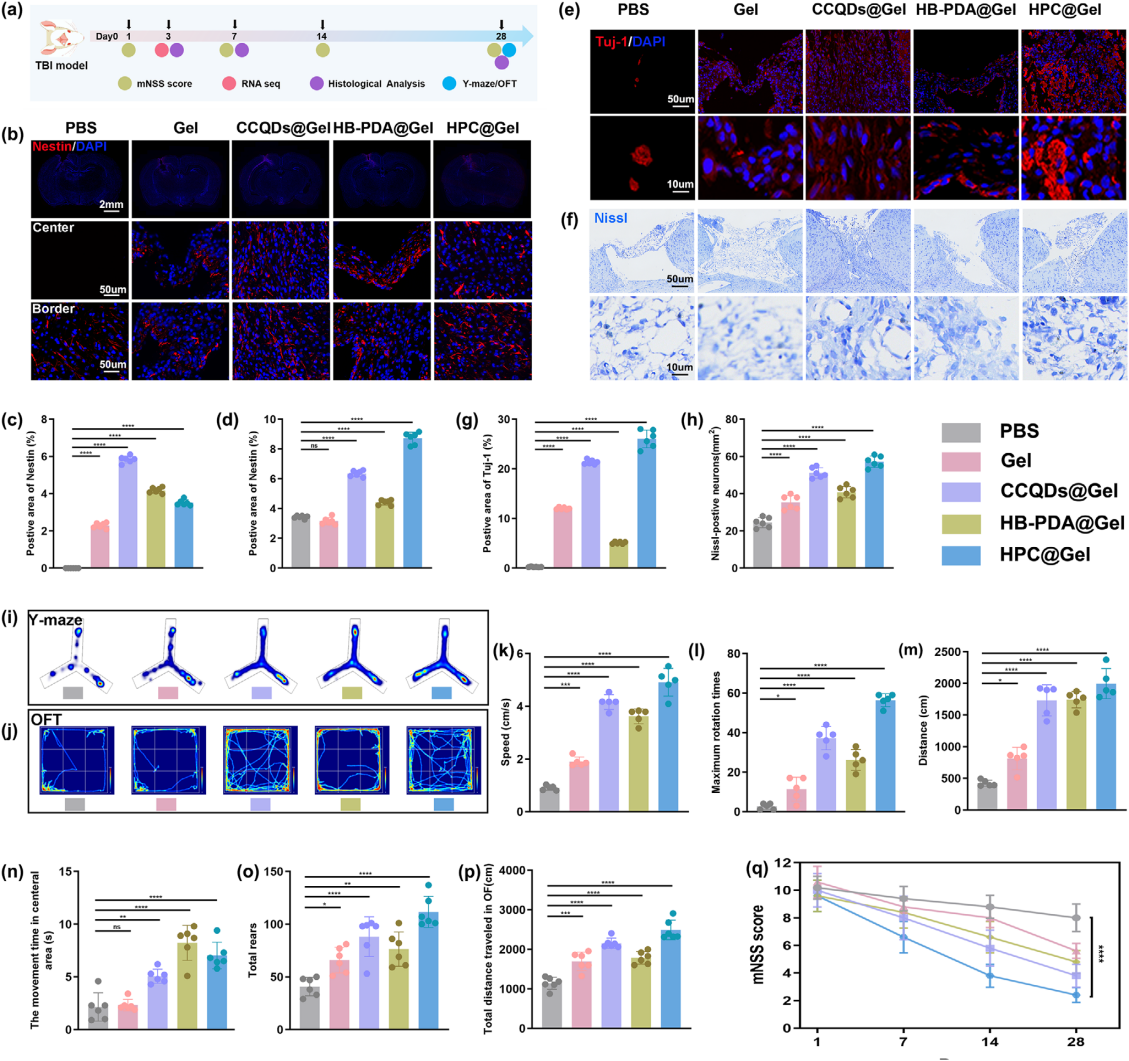
Bionic hydrogel system promotes post‐TBI neurogenesis and improves motor and cognitive functions. (a) Schematic illustration of the TBI animal experiment and hydrogel application. (b) Representative immunofluorescence images of the neural progenitor cell marker Nestin (red) in the lesion center and border at 7 days post‐TBI. Nuclei are stained with DAPI (blue). (c‐d) Quantitative analysis of the Nestin‐positive area in the lesion (c) center and (d) border at 7 days post‐TBI. Representative immunofluorescence images of Tuj‑1 in the lesion area at 7 days post‑TBI (e), and representative Nissl staining images at 28 days post‑TBI (f). Quantitative analysis of the Tuj‐1‐positive area (g) and the number of Nissl‐positive neurons (h). Representative tracking paths of rats in the (i) Y‐maze and (j) open‐field test (OFT) at 28 days post‐TBI. (k) Average speed of rats in the Y‐maze. (l) Maximum rotation times, assessing motor coordination. (m) Total distance traveled by rats in the Y‐maze. (n) The movement time in the central area during the OFT. (o) Total number of rears during the OFT. (p) Total distance traveled by rats during the OFT. (q) mNSS assessments on days 1, 7, 14, and 28 post‐TBI. Data are shown as mean ± SD based on six independent experiments. Data are shown as mean ± SD (*n* = 6 animals per group). For single‐time‐point comparisons, group differences were evaluated using one‐way ANOVA followed by Tukey's multiple‐comparison test. For mNSS analysis over time (q), statistical significance was analyzed using two‐way ANOVA followed by an appropriate multiple‐comparison test. Significance levels are indicated as ^*^ *p* < 0.05, ^**^ *p* < 0.01, ^***^ *p* < 0.001, ^****^ *p* < 0.0001.

Based on the favorable in vitro performance of the biomimetic hydrogel system, we further established a TBI rat model to evaluate the therapeutic potential of HPC@Gel in vivo. As shown in Figure 5a, animals were randomly assigned to five groups (PBS, Gel, CCQDs@Gel, HB‐PDA@Gel, and HPC@Gel), and the corresponding hydrogels were implanted at the injury site immediately after TBI induction. Systemic evaluations were performed at multiple time points after surgery (days 3, 7, and 28).

After TBI, neuronal loss and structural disruption in the injured area are the main causes of irreversible neurological deficits. Thus, stimulating endogenous neural regeneration is considered a key strategy for promoting brain repair and functional recovery [38, 39]. To investigate whether the biomimetic hydrogel system can facilitate endogenous regeneration, we assessed neural stem/progenitor cells (NSPCs) and newly generated neurons in the core and surrounding areas at early (day 7) and late (day 28) post‐injury stages. We first examined Nestin, a marker of NSPCs. On day 7, compared with the PBS group, all treatment groups exhibited a marked increase in Nestin‐positive cells in the peri‐injury region (Figure 5b). Interestingly, even the Gel group alone enhanced NSPC proliferation, suggesting that the physical/mechanical properties of the biomimetic hydrogel itself provide a pro‐regeneration “niche” independent of bioactive nanoparticles, emphasizing the crucial role of providing mechanical compatibility and supportive physical niches for endogenous repair mechanisms. This finding was consistent with our previous results [40]. Comparison of Nestin expression in the central versus peripheral regions further indicated that neural regeneration initially arose in the peri‐injury zone and gradually migrated into the injury core over time (Figure 5c,d). By day 28, the persistent presence of Nestin‐positive cells suggested that the hydrogel system maintained a long‐term pro‐neuroregenerative microenvironment (Figure S12) [41].

To assess neuronal differentiation, we evaluated Tuj‐1, a marker of immature neurons [42]. On day 7, only a few Tuj‐1–positive cells were detected in the PBS group, whereas all treatment groups showed significant neuronal differentiation, with the strongest effect observed in the HPC@Gel group (Figure 5e,g). This indicates that the hydrogel system promoted early neuronal regeneration. To further determine the long‐term survival and integration of newly formed neurons, we quantified Nissl‐positive neurons on day 28 [43]. The results revealed that neuron numbers were markedly higher in the treatment groups compared with PBS controls (Figure 5f,h). Together, these findings demonstrate that the biomimetic hydrogel system activates NSPCs, promotes their proliferation, guides their neuronal differentiation, and supports the long‐term survival of new neurons—thereby providing a robust cellular foundation for structural repair and functional recovery after TBI [40].

Behavioral analyses were performed to determine whether the observed histological improvements translated into functional recovery [38]. The Y‐maze test, performed on day 28, was used to assess spatial learning and memory. Compared with PBS‐treated rats, which displayed prolonged retention in the maze and reduced mobility, all hydrogel‐treated groups showed enhanced activity. Quantitative analysis of maximum rotation frequency, total distance traveled, and average speed confirmed significant improvements, with HPC@Gel exhibiting the most pronounced effect (Figure 5i,k–m). These results suggest that HPC@Gel markedly improves spatial learning and memory in TBI rats.

Animals’ locomotion, exploratory behavior, and indicators of anxiety were measured through the open field test (OFT) [44]. PBS rats demonstrated typical anxiety‐like behavior, remaining along the periphery and avoiding the central area. By contrast, CCQDs@Gel and HPC@Gel groups exhibited significantly increased exploration, with greater time spent in the center and reduced peripheral preference (Figure 5j). Quantitative analysis further showed that the HPC@Gel group had the longest central area duration and highest locomotor activity (Figure 5n–p), suggesting that treatment effectively reduced anxiety and enhanced exploratory motivation.

Neurological performance was systematically assessed with the modified neurological severity score (mNSS) on days 1, 7, 14, and 28 following surgery [45]. Across the four weeks, all treatment groups exhibited progressively lower scores than PBS controls, with the HPC@Gel group showing the most significant improvements, surpassing the Gel, CCQDs@Gel, and HB‐PDA@Gel groups (Figure 5q).

In summary, both histological and behavioral assessments consistently demonstrate that HPC@Gel showed stronger therapeutic effects than Gel alone, indicating that its efficacy was not solely attributable to the mechanical support provided by the hydrogel matrix. HPC@Gel not only promotes NSPC activation, proliferation, and neuronal regeneration but also significantly alleviates neurological deficits, highlighting its strong therapeutic potential for TBI treatment.

### 2.6 The Neuroprotective and Neuroinflammatory Regulatory Effects of Bionic Gel Systems In Vivo

Collectively, the findings demonstrate that the biomimetic hydrogel system can facilitate neural repair and regeneration. To further elucidate its reparative mechanism, we investigated alterations in the injured microenvironment, focusing on neuronal hypoxia, apoptosis, and neuroinflammation. Previous studies have shown that expression of HIF‐1α markedly increases after TBI, accompanied by extensive neuronal apoptosis [46]. Thus, improving the local injury microenvironment, including hypoxia‐associated stress, is important for mitigating neuronal apoptosis [47].

**FIGURE 6:**
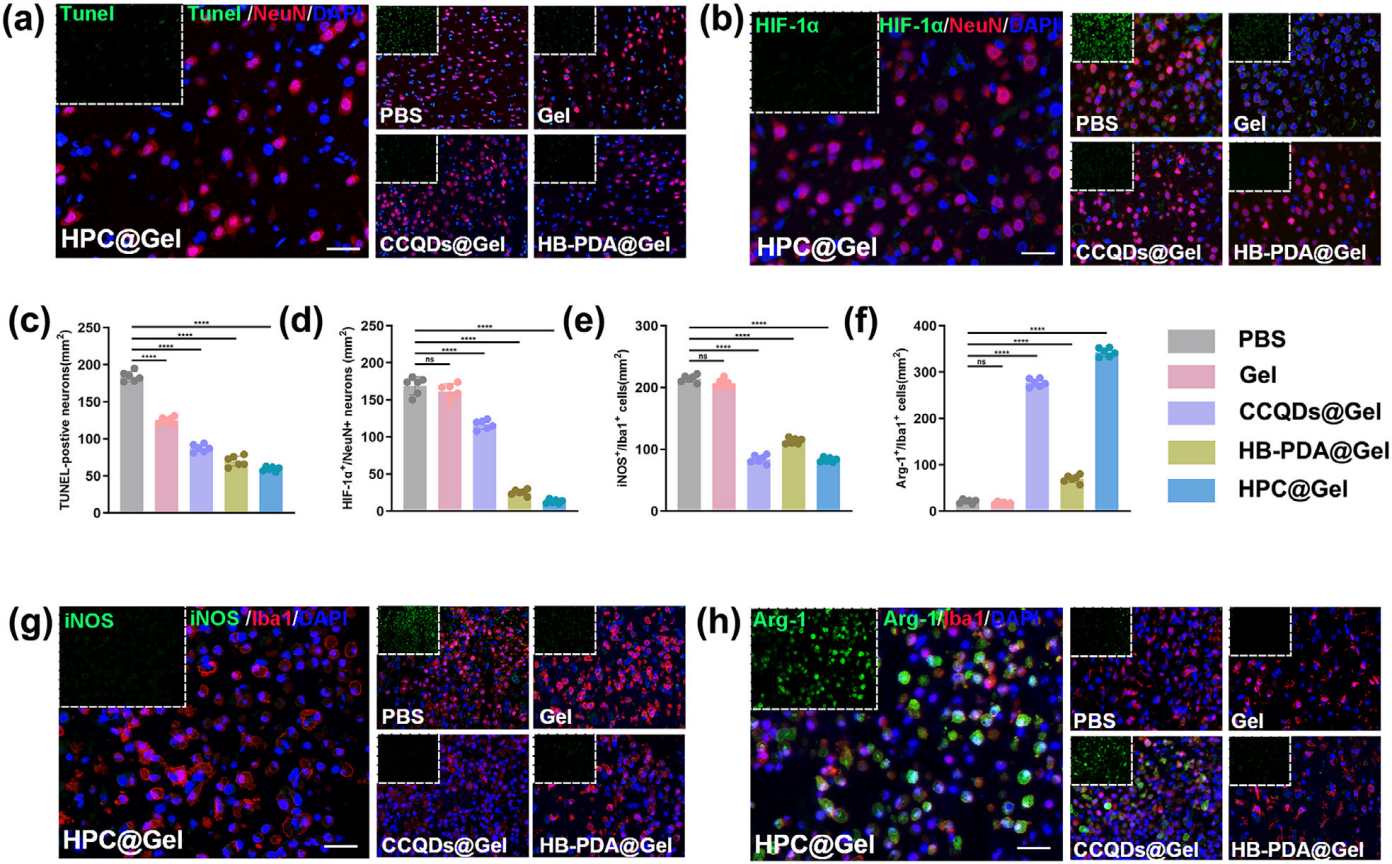
The biomimetic hydrogel system confers in vivo neuroprotection at 3 days post‐TBI. (a) Representative immunofluorescence images showing apoptotic cells (TUNEL, green) and neurons (NeuN, red) in the pericontusional cortex. (b) Representative immunofluorescence images showing hypoxic cells (HIF‐1α, green) and neurons (NeuN, red). (c) Quantification of TUNEL‐positive neurons. (d) Quantification of HIF‐1α‐positive neurons. Quantification of (e) iNOS‐positive microglia cells and (f) Arg‐1‐positive microglia cells. Representative immunofluorescence images showing (g) iNOS (green)/Iba‐1 (red) and (h) Arg‐1(green) and Iba‐1 (red). Data are presented as mean ± SD (*n* = 6 animals per group). Statistical significance was analyzed using one‐way ANOVA followed by Tukey's multiple‐comparison test. ^*^ *p* < 0.05, ^**^ *p* < 0.01, ^***^ *p* < 0.001, ^****^ *p* < 0.0001.

As shown in Figure 6a,c, compared with the PBS group, TUNEL and NeuN co‐labeling revealed that treatment with the hydrogel markedly reduced neuronal apoptosis in the injured region, supporting the neuroprotective effect of HPC@Gel in TBI. Besides, the number of HIF‐1α–positive neurons in the HB‐PDA@Gel and HPC@Gel groups was significantly reduced, indicating that the HB‐containing hydrogel effectively alleviated hypoxia‐associated neuronal stress in vivo (Figure 6b,d) [48]. Furthermore, this phenomenon of reducing neuronal hypoxia and improving neuronal apoptosis became more significant on the seventh day after the operation (Figure S13).

Following TBI, microglia remain persistently activated, predominantly assuming the pro‐inflammatory M1 phenotype, which aggravates secondary injury and contributes to long‐term neurological deficits. In contrast, the anti‐inflammatory M2 phenotype exerts neuroprotective functions. Therefore, suppressing excessive microglial activation and promoting M2 polarization are essential for restoring the injured microenvironment [49]. Using Iba‐1 staining, we evaluated microglial activation and neuroinflammation at 3 and 7 days post‐TBI. On day 3, the PBS group exhibited elevated pro‐inflammatory markers (iNOS) and reduced anti‐inflammatory markers (Arg‐1). In contrast, treatment with CCQDs@Gel, HB‐PDA@Gel, and HPC@Gel significantly decreased pro‐inflammatory (iNOS+) microglia and increased anti‐inflammatory (Arg‐1+) cells (Figure 6e–h). This favorable phenotypic shift persisted through day 7, indicating that the biomimetic hydrogel continuously suppresses excessive microglial activation while maintaining a balance between pro‐ and anti‐inflammatory responses (Figure S14).

In conclusion, the biomimetic hydrogel system substantially optimizes the post‐TBI microenvironment by alleviating neuronal hypoxia, inhibiting apoptosis, and modulating neuroinflammation, thereby achieving robust neuroprotection.

### 2.7 Biomimetic Composite Gel Reduces S100A8 Expression and Exhibits High Biocompatibility in Vivo, With Underlying Mechanisms Uncovered Through Transcriptomics

**FIGURE 7:**
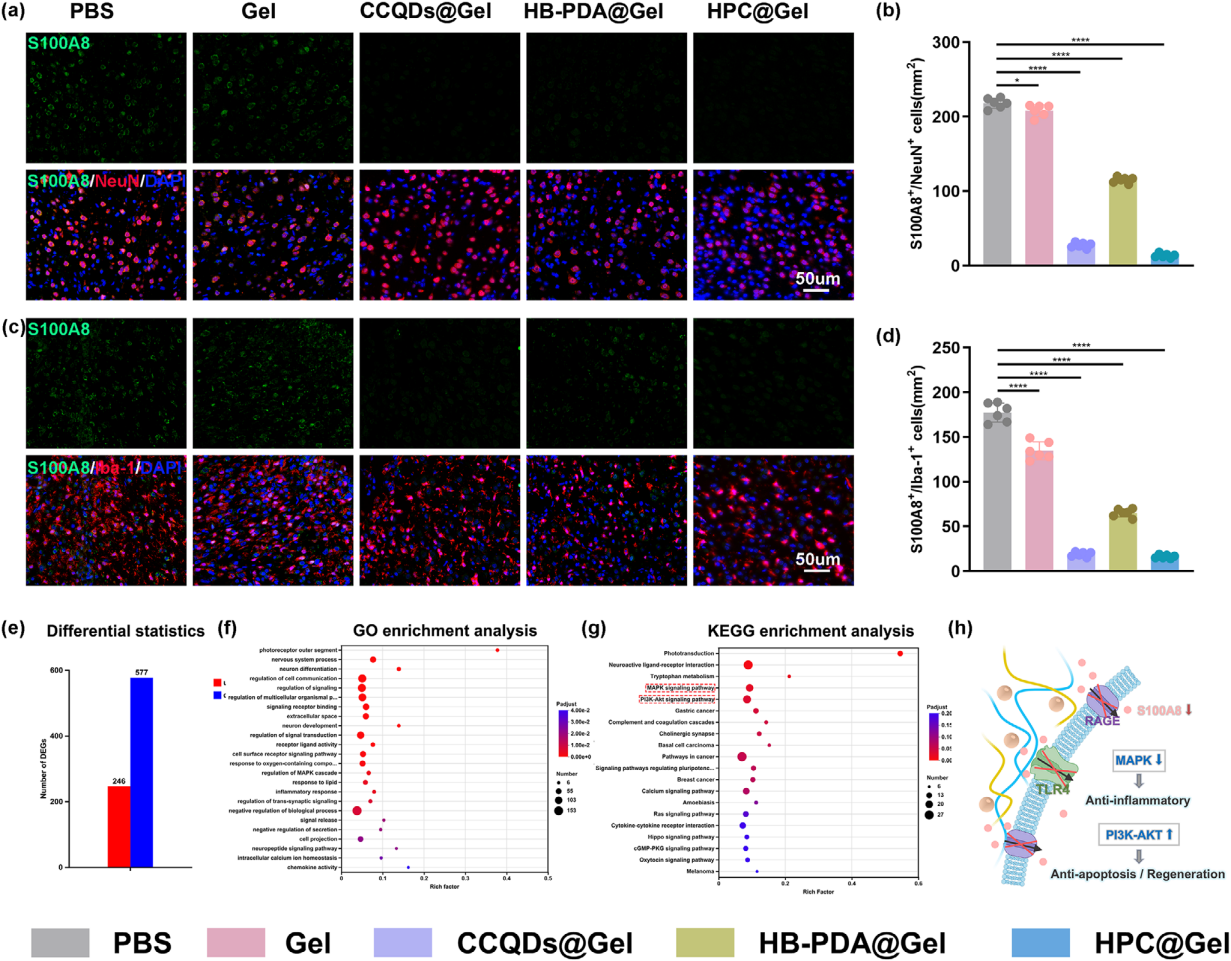
Bionic hydrogel system reduces S100A8 expression and transcriptome sequencing reveals potential signaling pathways. Representative immunofluorescence images of S100A8 (green) co‐stained with (a) NeuN (red) and (c) Iba‐1 (red) in the TBI lesion area. Nuclei are counterstained with DAPI (blue). Quantitative analysis of the number of (b) S100A8+/NeuN+ cells and (d) S100A8+/Iba‐1+ cells. For immunofluorescence quantification, data are presented as mean ± SD (*n* = 6 animals per group). Statistical significance was analyzed using one‐way ANOVA followed by Tukey's multiple‐comparison test. (e) Bar chart of DEGs identified by transcriptome sequencing, comparing the HPC@Gel group with the PBS group. (f) GO enrichment analysis of the identified DEGs. (g) KEGG pathway enrichment analysis of the identified DEGs. (h) Schematic diagram illustrating the potential signaling pathway modulated by the bionic hydrogel. Data are presented as mean ± SD (*n* = 6 animals per group). For RNA‐seq, *n* = 3 biologically independent samples per group. ^*^ *p* < 0.05, ^**^ *p* < 0.01, ^***^ *p* < 0.001, ^****^ *p* < 0.0001.

As a DAMP, S100A8 mediates neuroimmune crosstalk by activating microglial inflammatory pathways, thereby exacerbating the TBI microenvironment. S100A8 serves as both an indicator and a therapeutic target, consistent with our observation of its significant upregulation during TBI (Figure 3b) [50, 51]. To further evaluate the regulatory effect of the hydrogel, we examined S100A8 expression in microglia and neurons by immunofluorescence at 3 and 7 days post‐injury. Compared with the PBS group, the HPC@Gel group exhibited markedly suppressed S100A8 expression in both neurons and microglia, demonstrating that HPC@Gel effectively reduces this inflammatory mediator in the injured tissue (Figure 7a–d and Figure S15).

To explore biological processes and signaling changes associated with HPC@Gel treatment after TBI, we performed transcriptome sequencing on brain tissue from the PBS group and the HPC@Gel group three days post‐injury. A total of 823 differentially expressed genes (DEGs) were identified, comprising 246 upregulated and 577 downregulated genes (Figure 7e). Detailed RNA‐seq quality‐control metrics, including sequencing depth and sample‐level quality parameters, are provided in Table S1 and Figure S16. GO and KEGG enrichment analyses were carried out to investigate the functional and pathway associations of the detected genes. GO enrichment suggested that these DEGs were associated with vascular function (GO:0043114), immune regulation (GO:0030217, GO:0008009), and neural development (GO:0097485, GO:0007399) (Figure 7f). KEGG enrichment further revealed significant involvement of the PI3K‐Akt and MAPK signaling pathways (Figure 7g). Activation of the PI3K‐Akt pathway is known to reduce neuronal apoptosis and enhance neural regeneration [52, 53]. In contrast, inhibition of MAPK signaling is associated with reduced M1 microglial activation and alleviation of neuroinflammation [54]. Previous studies have demonstrated that S100A8 mediates destructive neuronal‐microglial crosstalk through RAGE and TLR4 on microglia, thereby triggering strong pro‐inflammatory responses [37]. Consistent with these findings, our earlier experiments confirmed that HPC@Gel alleviates neuroinflammation and exerts neuroprotective effects by disrupting S100A8‐mediated neuronal‐microglial interactions. However, because PI3K‐Akt and MAPK signaling are responsive not only to oxygen status but also to oxidative stress and inflammatory regulation, the observed transcriptomic changes are more consistent with broad biological modulation in the injured tissue than with a specific oxygen‐delivery mechanism. In particular, the downregulation of MAPK‐associated genes is consistent with reduced stress‐ and inflammation‐related signaling after HPC@Gel treatment (Figure 7h).

Histological examination using hematoxylin‐eosin (HE) staining in 28 days post‐TBI revealed no pathological abnormalities in the heart, liver, spleen, lungs, or kidneys of the experimental groups, indicating the absence of tissue toxicity or structural damage (Figure S17a). Venous blood biochemical assessment further demonstrated that the levels of alanine aminotransferase (ALT), aspartate aminotransferase (AST), creatinine (CREA), and blood urea nitrogen (BUN) in the experimental groups remained within the normal range and did not differ significantly from the values measured in the PBS group. (Figure S17b–e). Taken together, these findings suggest that the biomimetic gel system exhibits favorable biocompatibility and does not induce apparent systemic toxicity in vivo.

## 3 Conclusion

This study developed a multifunctional biomimetic hydrogel system (HPC@Gel) for the treatment of TBI. Rather than acting through isolated suppression of a single inflammatory mediator, the system is designed to concurrently alleviate tissue hypoxia and attenuate S100A8‐associated neuroimmune crosstalk, thereby remodeling the post‐injury microenvironment to support neural repair. Based on a brain tissue–mimicking gel matrix, integrating the synergistic function of oxygen release and anti‐inflammatory activity, the system alleviates tissue hypoxia and suppresses detrimental neuroimmune crosstalk mediated by the key inflammatory factor S100A8, in addition to providing regenerative niches. Experimental findings demonstrated that this strategy not only protects neurons and limits excessive microglial activation but also markedly promotes the growth and maturation of endogenous neural stem cells in animal models, leading to significant improvement in neurological function. In summary, this work establishes a novel therapeutic strategy for TBI by demonstrating that a biomimetic hydrogel system, co‐delivering an oxygen carrier and an anti‐inflammatory agent, can simultaneously ameliorate hypoxia and disrupt detrimental S100A8‐mediated neuroimmune crosstalk. This dual‐pronged approach effectively remodels the post‐injury microenvironment to foster robust neural regeneration and functional recovery.

## 4 Experimental Section

### 4.1 Materials

Hyaluronic acid(HA, 80kDa) from Freda (China). Curcumin (≥99.8%) from Sinopharm (China). The following chemicals were obtained from Sigma–Aldrich (USA): hemoglobin (HB), sodium periodate, methacrylic anhydride (MA, ≥98%), 2‐hydroxy‐4'‐(2‐hydroxyethoxy)‐2‐methylpropiophenone (I2959, 98%), and dopamine hydrochloride (DA·HCl, ≥99.8%). Tris‐HCl was obtained from Beyotime Biotechnology (China). Lipopolysaccharide (LPS) was purchased from Sigma–Aldrich (cat. no. L2880, USA). Dulbecco's Modified Eagle's medium (DMEM), trypsin‐EDTA solution (0.25%), Fetal bovine serum (FBS) was purchased from Gibco (USA). The primary antibodies against CD86, CD206, HIF‐1α, and S100A8 utilized for immunofluorescence staining were procured from Proteintech Group (China). S100A8, TNF‐α, and IL‐10 were quantitatively measured by an enzyme‐linked immunosorbent assay (ELISA) kit (Changzhou Chenjun Chemical Co., Ltd., China). The antifade mounting medium (with DAPI), live‐cell Caspase‐3 activity assay, Annexin V apoptosis detection kit, and CCK‐8 assay kit were obtained from Beyotime Biotechnology (China). The Alexa FluorTM 555‐ and 488‐conjugated secondary antibodies were provided by Invitrogen (USA). Paquinimod was purchased from MedChemExpress (USA). The antibodies and kits required for brain tissue section analysis, including the TUNEL Kit, LFB staining solution, Nissl staining solution, and antibodies against Iba‐1, NeuN, Nestin, S100A8, iNOS, TNF‐α, Tuj‐1, HIF‐1α, and MBP, as well as silver staining solution, were all purchased from Servicebio (China). The Sprague‐Dawley (SD) rats were provided by Chengdu DaShuo Biological Technology (China). Milli‐Q ultrapure water system (USA) was used for all experiments.

### 4.2 Synthesis of CCQDs

A mixed solution of curcumin and water (40 mg/mL) was transferred into a polytetrafluoroethylene‐lined autoclave and heated for 12 h at 180 °C. The resultant solution was centrifuged (12 000 rpm, 30 min) to remove large particles, followed by filtration through a 0.22 µm membrane. Subsequently, dialysis (MWCO 1 kDa) was performed to eliminate small molecular impurities. The purified CCQDs solution was lyophilized to obtain solid powder for long‐term storage.

### 4.3 Synthesis of HPC

HB and DA·HCl were mixed at a mass ratio of 20:1 and reacted in Tris‐HCl solution (pH 8.5) at 4°C for 30 min. Thereafter, CCQDs (1 mg/mL) were added and the mixture was stirred for an additional 210 min. Unreacted monomers were removed using an ultrafiltration tube (30 kDa). The synthesis of HB‐PDA followed the same procedure except that CCQDs were not introduced.

### 4.4 Preparation of HPC@Gel

First, bifunctionalized HA was synthesized. Sodium periodate was added to a solution of HA (1 wt%) in RO water and reacted in the dark for 24 h, followed by dialysis and lyophilization to obtain aldehyde‐modified HA (AHA). Subsequently, AHA (1 wt%) was dissolved in PBS, followed by the addition of MA (3.2 mL). The pH was adjusted to 8 with 1 M NaOH, and the mixture was allowed to react at 4°C for 24 h. After dialysis and freeze‐drying, methacrylate‐grafted AHA (AHAMA) was obtained.

AHAMA (0.8 wt%) and Col (0.8 wt%) were dissolved in PBS containing I2959 (0.5 wt%) and 0.5 M acetic acid, respectively. The two solutions were mixed at equal volumes and the pH was adjusted to 7–7.4. The mixture was allowed to undergo self‐assembly at 37°C for 30 min, followed by UV irradiation (365 nm, 3 W/cm^2^) for 30 s to yield the hydrogel scaffold (Gel). During gelation, CCQDs, HB‐PDA, or HPC were respectively introduced to obtain CCQDs@Gel, HB‐PDA@Gel, and HPC@Gel.

### 4.5 Physicochemical Characterization

The morphology of the samples was observed using SEM (Hitachi, Japan). Lattice spacing was determined by TEM (Thermo Fisher Scientific, USA). Functional groups were analyzed by FTIR (Thermo Fisher Scientific, USA) with a resolution of 4 cm^−^^1^ within a scanning range of 400–4000 cm^−^^1^. The UV–vis absorbance spectra were recorded using a spectrophotometer (Evolution Pro, Thermo Scientific) in the wavelength range of 200–700 nm. The chemical structures were characterized by ^1^H NMR at 600 MHz (Bruker, Switzerland). For degradation and swelling studies, hydrogels were immersed in type I collagenase solution and PBS at 37°C for 72 h. The degradation rate and swelling ratio were calculated using the following formulas: Degradation rate = (W_0_ – Wt)/W_0_ × 100%, Swelling ratio = (Wt—W_0_)/W_0_ × 100%, where W_0_ and Wt represent the initial mass and the mass at each time point, respectively. For colloidal stability analysis, HPC nanoparticles were dispersed in PBS and incubated at 37°C. Hydrodynamic diameter and zeta potential were measured at days 0, 3, 5, and 7 using dynamic light scattering.

### 4.6 Mechanical Performance Testing

Hydrogels with defined dimensions (diameter 10 mm, height 2 mm) were subjected to compression tests on a dynamic mechanical analyzer (DMA, TA‐Q800, USA). Young’ s modulus was derived from the linear segment slope of the stress‐strain curve at a 10%/min compression rate.

Cylindrical hydrogels (diameter 10 mm, height 2 mm) were tested on a rheometer (MCR‐302, Anton Paar Instruments) for stress relaxation behavior under 10% strain for 600 s. The normalized stress values were plotted to obtain stress relaxation curves.

### 4.7 In Vitro Drug Sustained‐Release Detection

The in vitro release profiles of CCQDs from HPC@Gel and CCQDs@Gel were investigated. Samples of each hydrogel were placed in phosphate‐buffered saline (PBS, pH 7.4) and incubated at 37°C with gentle shaking. The study was conducted using the sampling and replacement method to maintain sink conditions. 50% of the total volume of the release medium was sampled at predetermined time intervals (0, 1, 2, 4, 16, 24, 48, and 72 h) for subsequent analysis. Immediately after each withdrawal, an equal volume of fresh, pre‐warmed PBS was added back to the system to keep the total volume constant. The collected samples were filtered through a 0.22 µm membrane to remove any particulates. The concentration of CCQDs in the filtered release medium was quantified by HPLC. The cumulative percentage of released CCQDs was calculated for each time point, and the data were plotted to generate the drug release curves.

### 4.8 Antioxidant Activity Assays

DPPH radical scavenging assay: DPPH was dissolved in ethanol (100 µmol/L) and the absorbance at 517 nm was recorded (denoted as A_0_). Hydrogel samples (20 µL) were mixed with 200 µL of the above solution and incubated for 1 h at 37°C in the dark. The absorbance at 517 nm was recorded as A_1_. The scavenging activity was calculated as: Scavenging rate = (A_0_ – A_1_)/A_0_ × 100%.

The ABTS radical scavenging ability of the composite hydrogels was evaluated using a T‐AOC assay kit (Beyotime, China). Briefly, 200 µL of ABTS working solution was added to each well of a 96‐well plate. Hydrogel samples were then introduced and allowed to react for 5 min. The absorbance of the supernatant was subsequently measured at 734 nm using a microplate reader.

### 4.9 Oxygen Release Behavior

Oxygen‐saturated hydrogel samples were mixed with deoxygenated 0.9% NaCl solution, and the dissolved oxygen concentration in the solution was monitored using a dissolved oxygen meter (JPB‐607A, Shanghai Inesa Scientific Instrument Co., Ltd.). Measurements were terminated once a stable value was reached. The reaction system was maintained at 37 ± 0.2°C by a temperature controller.

### 4.10 Cell Culture

The BV2 (RRID: CVCL_0182) and HT22 (RRID: CVCL_0321) cell lines were purchased from Immocell Company (Xiamen, China). Both cell lines were verified to be free of mycoplasma contamination and authenticated using STR profiling. They were subsequently maintained in high‐glucose DMEM supplemented with 10% FBS and 1% penicillin–streptomycin at 37°C in a humidified incubator containing 5% CO_2_. For serum‐free stimulation experiments, BV2 cells were treated with LPS (1 µg/mL) for 24 h. After LPS stimulation, the LPS‐containing medium was removed and replaced with fresh complete medium before hydrogel treatment. Hypoxic conditions were established at 37°C with 5% CO_2_ and 1% O_2_.

### 4.11 CCK8 Cytotoxicity Assay

Cytotoxicity was assessed using the CCK8 assay kit according to the manufacturer's instructions. HT22 cells (5,000 per well) were seeded in 96‐well plates. CCQDs were added at final concentrations of 10, 25, 50, 100, 250, and 500 µg/mL, along with blank and control groups. Each condition was tested in triplicate. After 24 h, the appropriate concentration was determined using CCK8. Subsequently, cytotoxicity of the composite nanoparticle gel was evaluated on days 1, 3, and 5. Briefly, a 10% (v/v) CCK‐8 solution was added to each well. After incubation for 1 h, the absorbance was measured at a wavelength of 450 nm.

### 4.12 ElisaElisa

Commercial ELISA kits were utilized to quantify TNF‐α, IL‐10, and S100A8 levels in the culture supernatants following the manufacturer's recommended protocols. Supernatants were collected and centrifuged (3,000 rpm) for 10 min to remove impurities. Standard and sample wells were prepared: 50 µL of serially diluted standards or 10 µL of sample plus 40 µL of diluent were added to each well, with blank wells left untreated. Next, 100 µL of HRP‐conjugated detection antibody was added and incubated for 1 h at 37°C. After washing, 50 µL each of substrates A and B was added, followed by incubation at 37°C in the dark for 15 min. 50 µL of stop solution was added to terminate the reaction, and the absorbance was measured at 450 nm. A standard curve was generated using Excel, and sample concentrations were calculated accordingly.

### 4.13 Flow Cytometry

Cells were collected without EDTA digestion and centrifuged (1 000 rpm) for 3 min. After washing once with pre‐cooled PBS (4°C), cells were centrifuged again for 5 min. Following counting, 1 × 10^5^ cells were resuspended in 300 µL of 1×Binding Buffer. Annexin V‐FITC (5 µL) was added and incubated in the dark at room temperature for 15 min, followed by PI staining (10 µL, 5 min). Before acquisition, 200 µL of 1×Binding Buffer was added. Samples were analyzed using a BD flow cytometer.

### 4.14 Apoptosis Analysis

Apoptosis was evaluated with the Live‐Cell Apoptosis Detection Kit following the manufacturer's protocol. HT22 cells (2 × 10^4^ per well) were seeded in 48‐well chamber slides and allowed to adhere for 8 h. A blank group and a treatment group were established, and cells were cultured under hypoxic conditions for 24 h. Medium was removed, and cells were washed once with PBS. Each well received 100 µL of detection solution containing 97 µL Annexin V‐mCherry Binding Buffer, 2.5 µL Annexin V‐mCherry, and 0.5 µL GreenNuc Caspase‐3 substrate (1 mm). After incubation in the dark for 30 min at room temperature, apoptosis was observed using a confocal microscope (Zeiss LSM880).

### 4.15 Transwell

HT22 cells (3 × 10^4^ per well) were seeded in the lower chamber of 24‐well Transwell plates, and LPS‐activated BV2 cells (1 × 10^4^ per well) were seeded in the upper chamber with 0.4 µm pores. Both cell types were cultured in DMEM (10% FBS) under hypoxic conditions (37°C, 5% CO_2_, 1% O_2_). After 24 h, supernatants and cells were collected for ELISA and immunofluorescence analysis.

### 4.16 Immunofluorescence

Cells were cultured on 48‐well chamber slides. After washing with PBS (3×5 min), the cells were sequentially fixed with 4% paraformaldehyde (15 min), permeabilized with 0.2% Triton X‐100 for (5 min), and blocked with 10% goat serum for (30 min). They were then incubated with primary antibodies overnight at 4°C, followed by incubation with secondary antibodies for 1 h at room temperature in the dark. Slides were washed with PBS three times between steps. Finally, 5 µL of mounting medium containing DAPI was added, and samples were examined under a confocal microscope. Intracellular hypoxia was further assessed using the oxygen‐sensitive probe Ru(dpp)_3_(PF_6_)_2_. During the last 4 h of the hypoxia treatment, cells were incubated with 5 µm Ru(dpp)_3_(PF_6_)_2_ in the dark, followed by confocal imaging under identical acquisition settings for all groups. Fluorescence intensity was quantified as an indicator of intracellular hypoxia.

### 4.17 Construction and Sample Collection of the TBI Model

All animal procedures in this study were approved by the Experimental Animal Management Committee of Sichuan Province (Approval Number: SYXK(Sichuan): 2019–189) and were performed in strict accordance with the National Research Council's guidelines for the care and use of laboratory animals. A total of ninety‐six SPF‐grade rats (12 weeks old, 200–220 g) were randomly assigned into five groups: PBS group (*n* = 18 + 3), Gel group (n = 18), CCQDs@Gel group (*n* = 18), HB‐PDA@Gel group (*n* = 18), and HPC@Gel group (*n* = 18 + 3). In addition, a sham group was included for baseline histological comparison. Rats in the sham group underwent the same surgical exposure procedures without creation of the cortical cavity injury. Following anesthesia and fixation, a 5‐mm cranial window was drilled into the right parietal bone (2 mm posterior to the sagittal suture and 4 mm lateral to the midline) using a handheld cranial drill. After removal of the dura mater, a cylindrical cavity (2 mm in diameter, 2 mm in depth) was created with a mold. Following meticulous hemostasis, 100 µL PBS was injected into the TBI cavity in the PBS group, whereas the other groups received implantation of cylindrical hydrogel composites of identical dimensions. According to the experimental design, tissue samples were harvested at 3, 7, and 28 days post‐operation for subsequent analyses. No animals were excluded from the final analysis, and all reported sample sizes reflect the full experimental cohorts.

### 4.18 Immunofluorescence and Histopathological Staining

At 3, 7, and 28 days post‐surgery, the rats were perfused. Their brains were fixed in 4% paraformaldehyde for more than 24 h, followed by paraffin sectioning at a thickness of approximately 4 µm. Immunofluorescence analysis was performed to detect Iba‐1, NeuN, Nestin, S100A8, iNOS, TNF‐α, Tuj‐1, HIF‐1α, and MBP. The procedure included antigen retrieval, serum blocking, incubation with primary antibodies, incubation with secondary antibodies, DAPI counterstaining of nuclei, quenching of tissue autofluorescence, mounting with antifade medium, and image acquisition. In addition, TUNEL staining was performed to assess neuronal apoptosis, and Nissl staining was employed to evaluate pathological changes in brain neurons.

### 4.19 Neurological Function Assessment

To minimize environmental stress, all rats were habituated to the experimental environment and familiarized with the Y‐maze 3 days prior to behavioral testing. On day 28 post‐surgery, spontaneous alternation tests were conducted, with each rat monitored for 8 min, and data were analyzed using EthoVision XT software. Motor function was assessed on days 1, 7, 14, and 28 post‐surgery using the modified neurological severity score (mNSS). Following 24 h of environmental adaptation, an open‐field test was performed to assess locomotor activity and anxiety‐like behavior.

### 4.20 In Vivo Biocompatibility Analysis

On day 28 post‐surgery, the five major visceral organs, including the heart, liver, spleen, lungs, and kidneys, were harvested following perfusion. Pathological alterations were examined by hematoxylin and eosin (HE) staining. In addition, 2 mL of venous blood was collected to assess hepatic and renal function.

### 4.21 Transcriptome Sequencing and Analysis

On day 3 post‐surgery, brain tissues from the injury sites of the PBS group and the HPC@Gel group were collected. The samples were cut into ∼0.5 cm pieces, rinsed to remove surface blood, blotted dry with filter paper, and transferred into pre‐cooled RNase‐free cryotubes. The tissues were rapidly frozen in liquid nitrogen for 0.5 h and subsequently subjected to eukaryotic mRNA sequencing using a second‐generation high‐throughput sequencing platform. Enrichment analysis was performed using the Gene Ontology (GO) and Kyoto Encyclopedia of Genes and Genomes (KEGG) databases, respectively.

### 4.22 Statistical Analysis

All data are expressed as mean ± standard deviation (SD). Statistical analyses were performed using GraphPad Prism 8, FIJI, FlowJo_v10.8.1_CL, and Origin 2021 software. Data distribution was assessed for normality prior to parametric testing. For single‐time‐point comparisons among multiple groups, one‐way ANOVA followed by Tukey's multiple‐comparison test was used. For repeated measurements over time, two‐way ANOVA followed by the appropriate post hoc multiple‐comparison test was applied. The specific statistical methods used for each dataset are described in the corresponding figure legends.

## Author Contributions

**Peng Liu**: writing – review and editing, writing – original draft, formal analysis, data curation, conceptualization. **Shichao Jiang**: software, investigation. **Xiaoyang Wu**: writing – review and editing, writing – original draft, conceptualization, formal analysis. **Liangxue Zhou**: conceptualization, investigation, funding acquisition, writing – review and editing. **Chengheng Wu**: investigation, data curation, formal analysis. **Jie Ding**: visualization, validation. **Jing Sun**: validation, supervision. **Dan Wei**: visualization, supervision. **Yuyan Wang**: visualization, validation. **Kai Wu**: validation, software, supervision. **Hongsong Fan**: investigation, funding acquisition, conceptualization, writing – review and editing. **Gaowei Li**: validation, supervision, funding acquisition. **Xiaoyin Liu**: investigation, funding acquisition, formal analysis.

## Conflicts of Interest

The authors declare no conflicts of interest.

## Supporting information




**Supporting File**: advs76220‐sup‐0001‐SuppMat.doc.

## Data Availability

The data that support the findings of this study are available from the corresponding author upon reasonable request.
