# Supplementary material for: Biomimetic Hydrogel System Targeting S100A8 Centered Neuroimmune Crosstalk and Hypoxia Induced Neuronal Injury
Source: Adv Sci (Weinh). 2026 Jun 22:e76220. Online ahead of print. doi: 10.1002/advs.76220 (PMC13336702; doi:10.1002/advs.76220)
Supplement: Supplementary file 1 — Supporting File: advs76220‐sup‐0001‐SuppMat.doc. [file ADVS-9999-e76220-s001.doc]

Supporting Information

Biomimetic Hydrogel System Targeting S100A8 Centered Neuroimmune Crosstalk and Hypoxia Induced Neuronal Injury

Peng Liu ^a,#^, Xiaoyang Wu ^b,#^, Xiaoyin Liu ^a^, Yuyan Wang ^b^, Shichao Jiang ^b^, Kai Wu ^b^, Gaowei Li ^a^, Jie Ding ^b^, Chengheng Wu ^b^, Dan Wei ^b^, Jing Sun ^b^, Hongsong Fan ^b^,*, and Liangxue Zhou ^a,c^,*

^a^ Department of Neurosurgery, West China Hospital, West China Medical School, Sichuan University, Chengdu, Sichuan 610041, China

^b^ National Engineering Research Center for Biomaterials, College of Biomedical Engineering, Sichuan University, Chengdu, Sichuan 610064, China

^c^ Department of Neurosurgery, NHC Key Laboratory of Nuclear Technology Medical Transformation (Mianyang Central Hospital) School of Medicine, University of Electronic Science and Technology of China, Mianyang, Sichuan 621000, China

^#^ Peng Liu and Xiaoyang Wu are co-first authors of this article.

*Corresponding author:

Hongsong Fan, E-mail: hsfan@scu.edu.cn

Liangxue Zhou, E-mail: zhlxlll@163.com


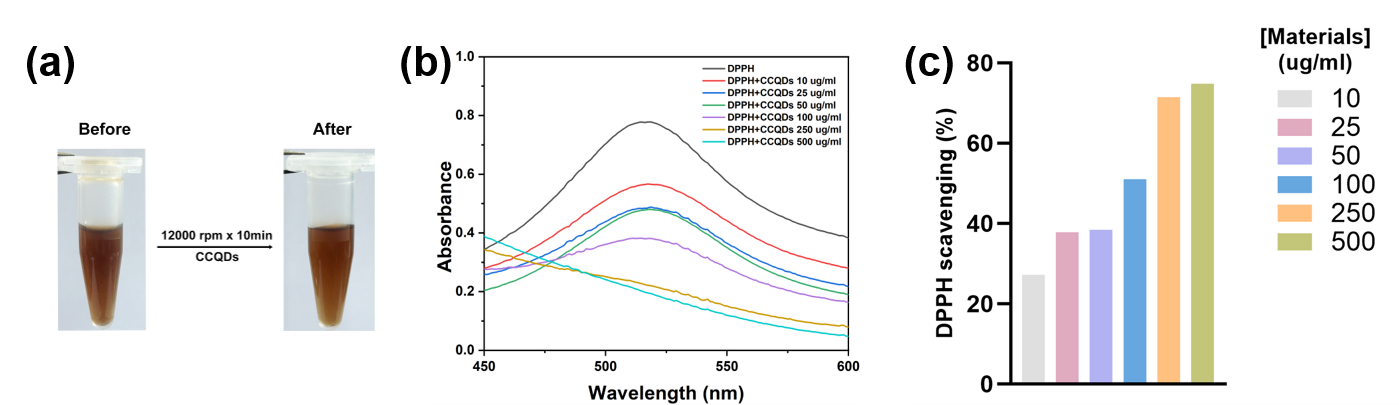


Figure S1. (a) Digital photographs of CCQDs before and after centrifugation, showing the excellent water dispersibility of CCQDs; (b) UV-Vis spectra of CCQDs for DPPH radical scavenging; (c) DPPH radical scavenging rates of CCQDs. Data are presented as mean ± SD from n = 3 independent experiments.


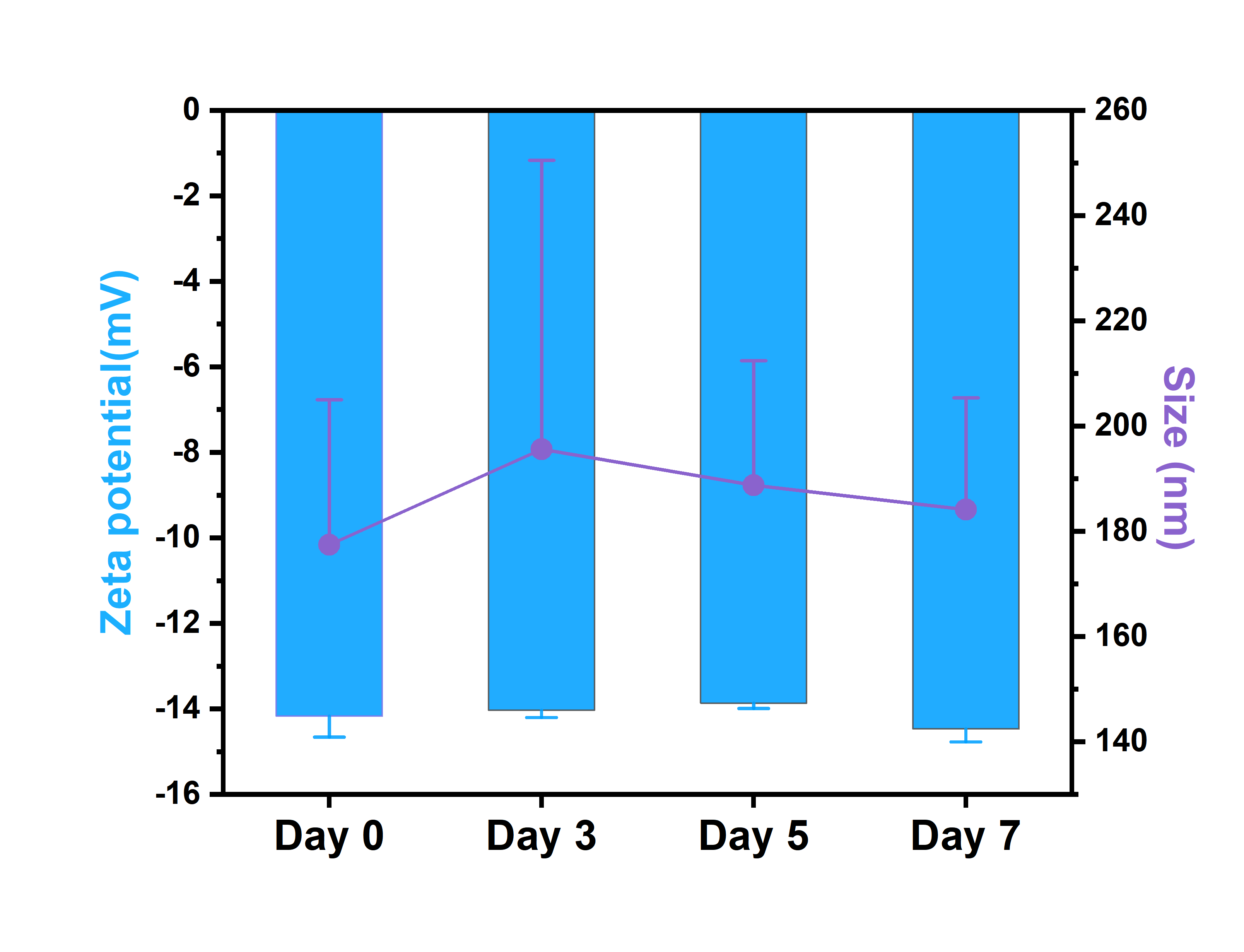


Figure S2. Colloidal stability of HPC nanoparticles in PBS over 7 days. Hydrodynamic diameter and zeta potential of HPC nanoparticles after incubation in PBS for 0, 3, 5, and 7 days. No significant changes were observed over the tested period, indicating good colloidal stability of HPC under physiological buffer conditions. Data are presented as mean ± SD from n = 3 independent experiments.


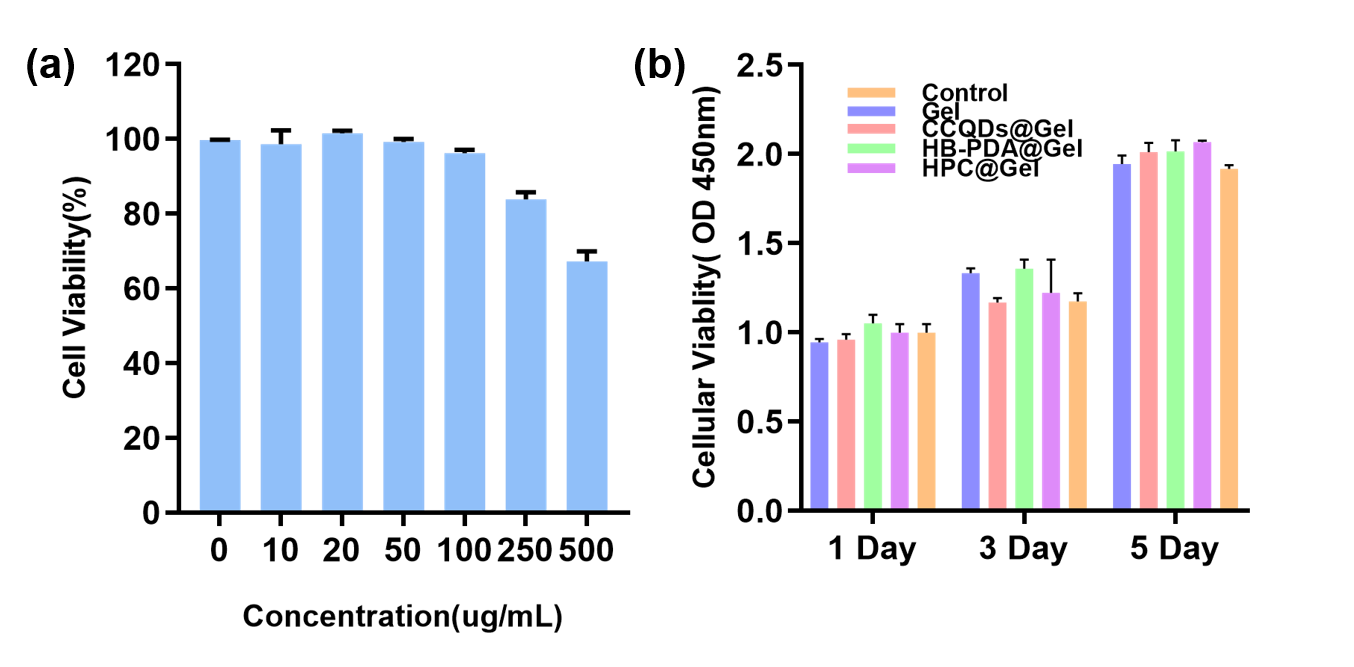


Figure S3. CCK-8 assay of (a) CCQDs at different concentrations and (b) composite hydrogel scaffolds in various groups. Data are presented as mean ± SD from n = 3 independent experiments.


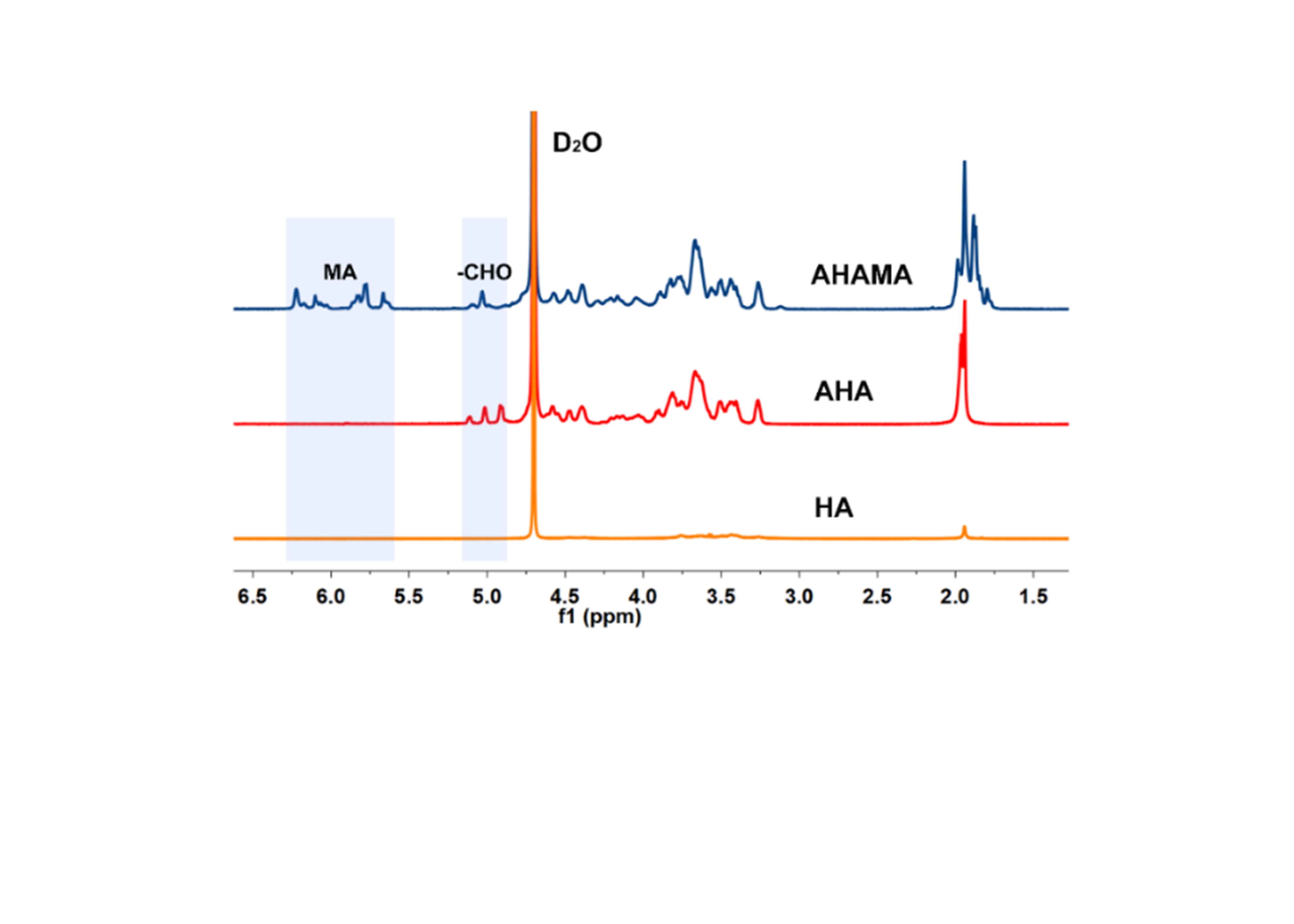


Figure S4. ^1^H NMR spectra of HA, AHA, and AHAMA.


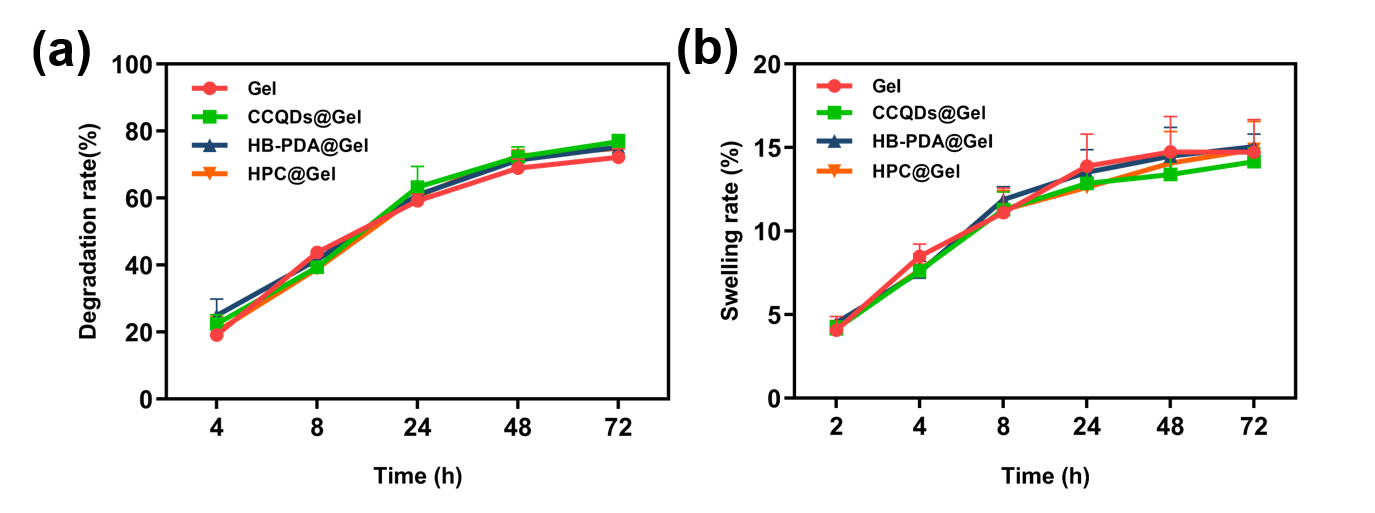


Figure S5. (a) Degradation rate and (b) swelling ratio of hydrogel scaffolds in different groups. Data are presented as mean ± SD from n = 3 independent experiments.


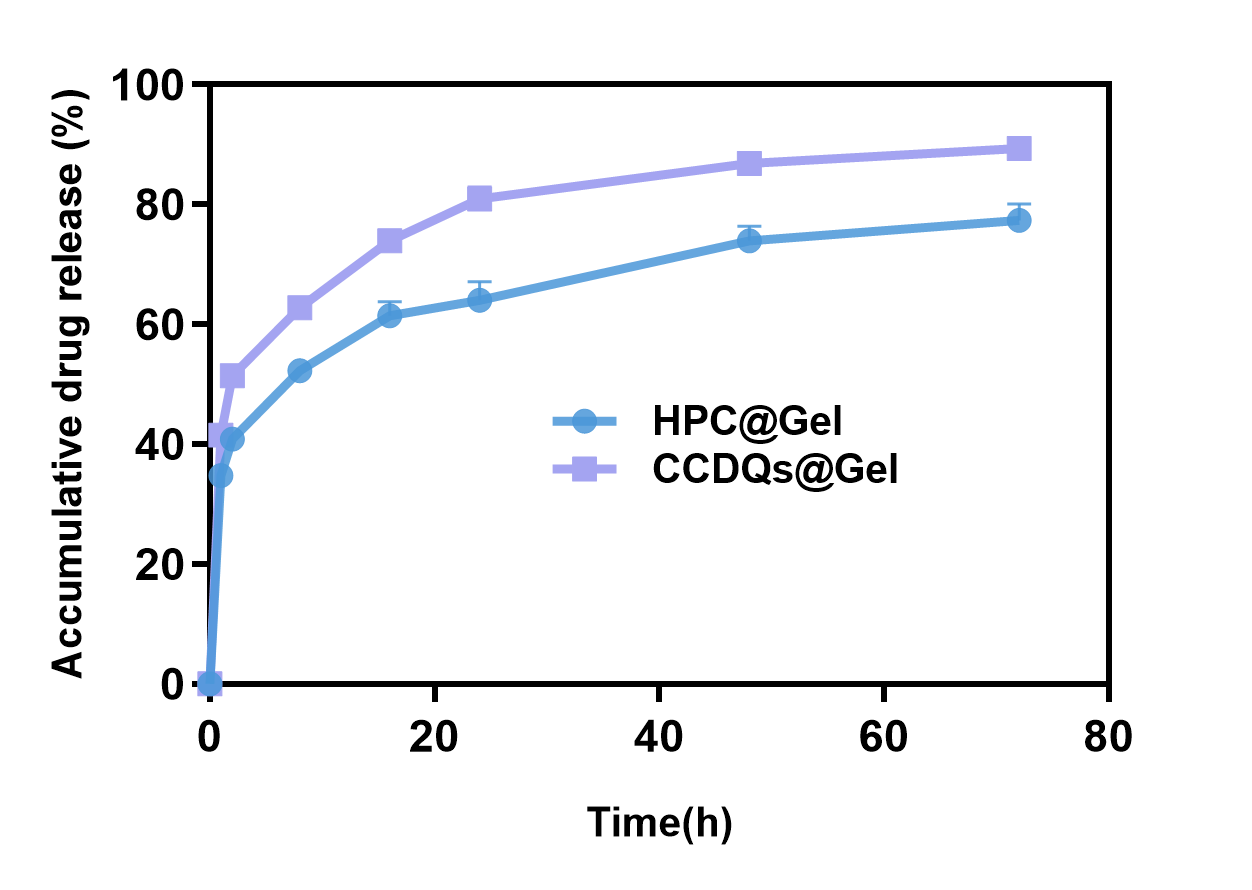


Figure S6. Curcumin release profiles of HPC@Gel and CCQDs@Gel. Data are presented as mean ± SD from n = 3 independent experiments.


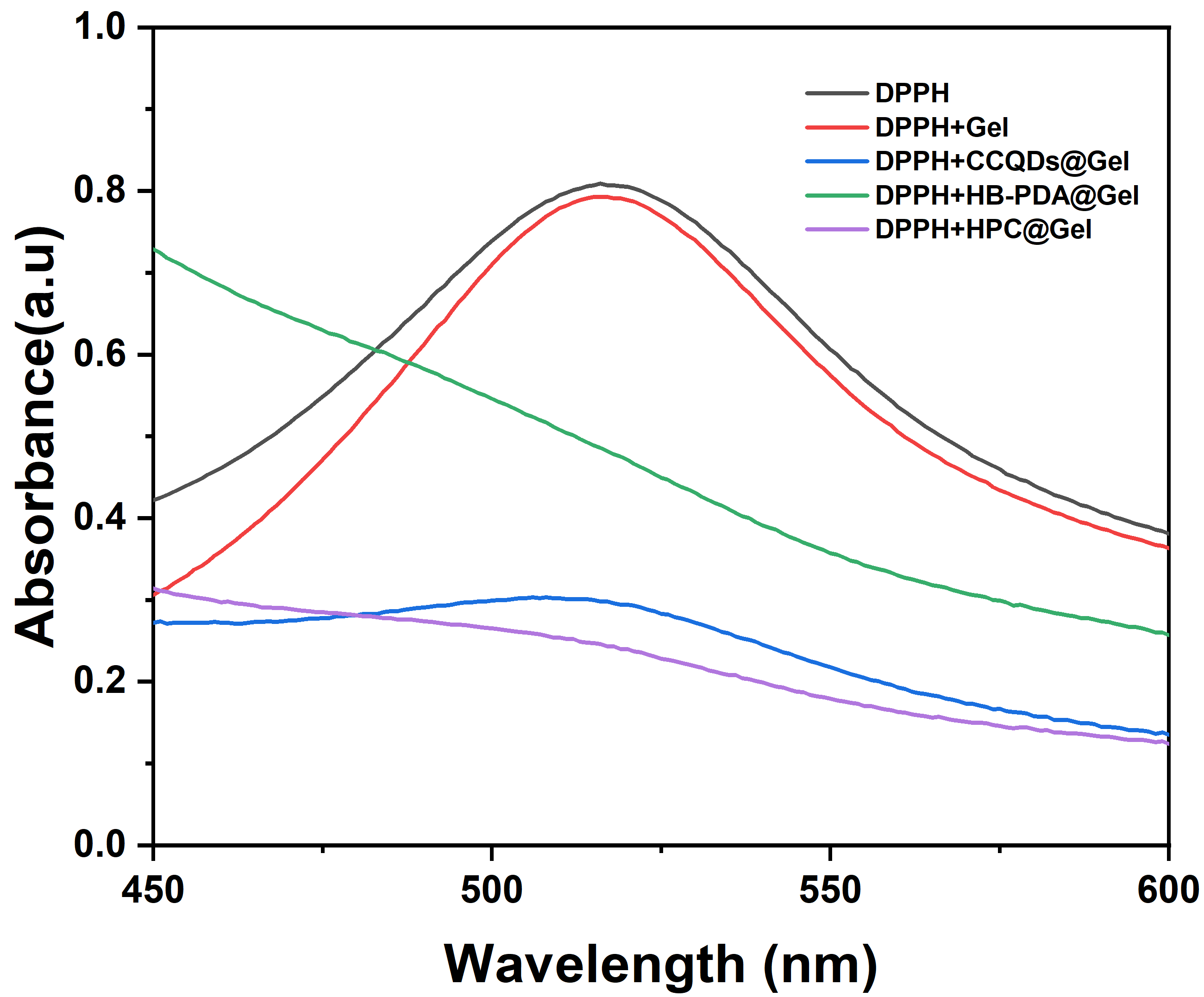


Figure S7. UV-Vis spectra of hydrogel scaffolds in different groups (DPPH scavenging assay).


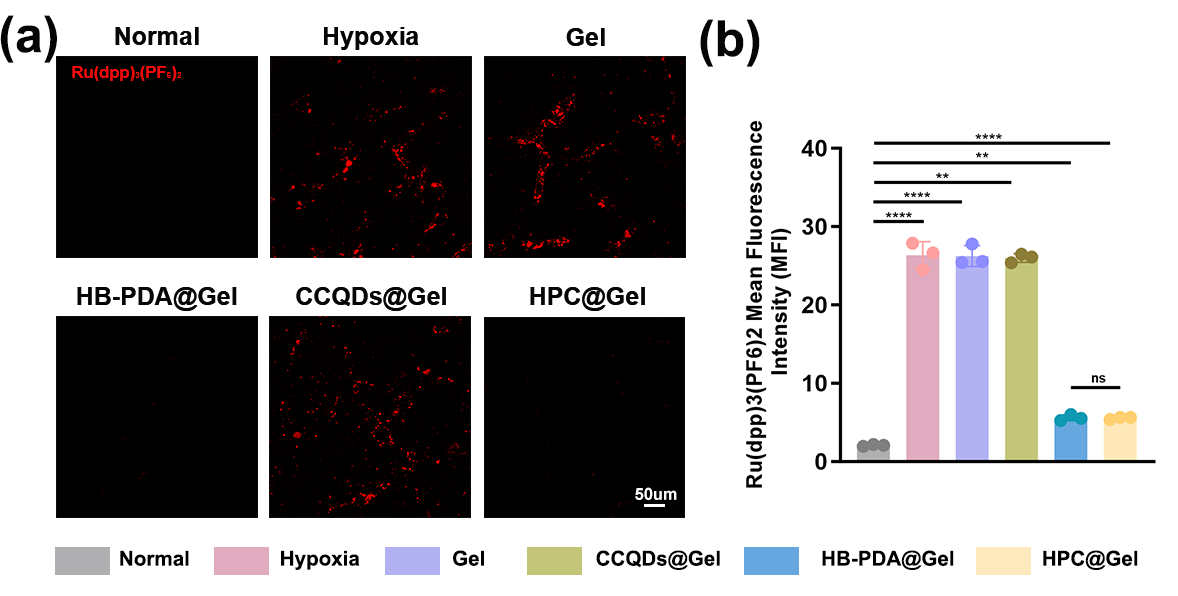


Figure S8. Direct assessment of intracellular hypoxia using Ru(dpp)3(PF6)2 staining. HT22 cells under different treatment conditions were incubated with the oxygen-sensitive probe Ru(dpp)3(PF6)2, and intracellular hypoxia was evaluated by confocal microscopy. Representative fluorescence images (a) and quantitative analysis of fluorescence intensity (b) are shown. Data are presented as mean ± SD from n = 3 independent experiments. Statistical significance was analyzed using one-way ANOVA followed by Tukey’s multiple-comparison test. ns, no significant difference; **p < 0.01; ***p < 0.001; ****p < 0.0001.


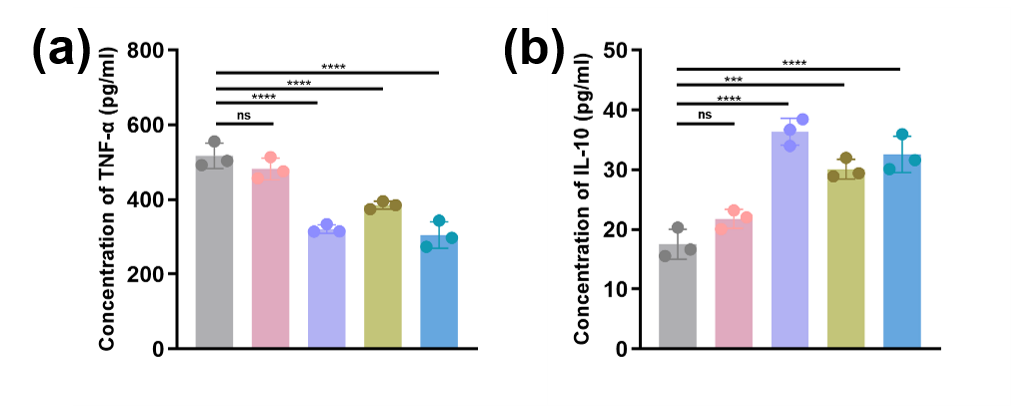


Figure S9. ELISA quantification of the TNF-α (a) and the IL-10 (b) in the supernatant. Data are presented as mean ± SD from n = 3 independent experiments. Statistical significance was analyzed using one-way ANOVA followed by Tukey’s multiple-comparison test. ns, no significant difference; *p < 0.05; **p < 0.01; ***p < 0.001; ****p < 0.0001.


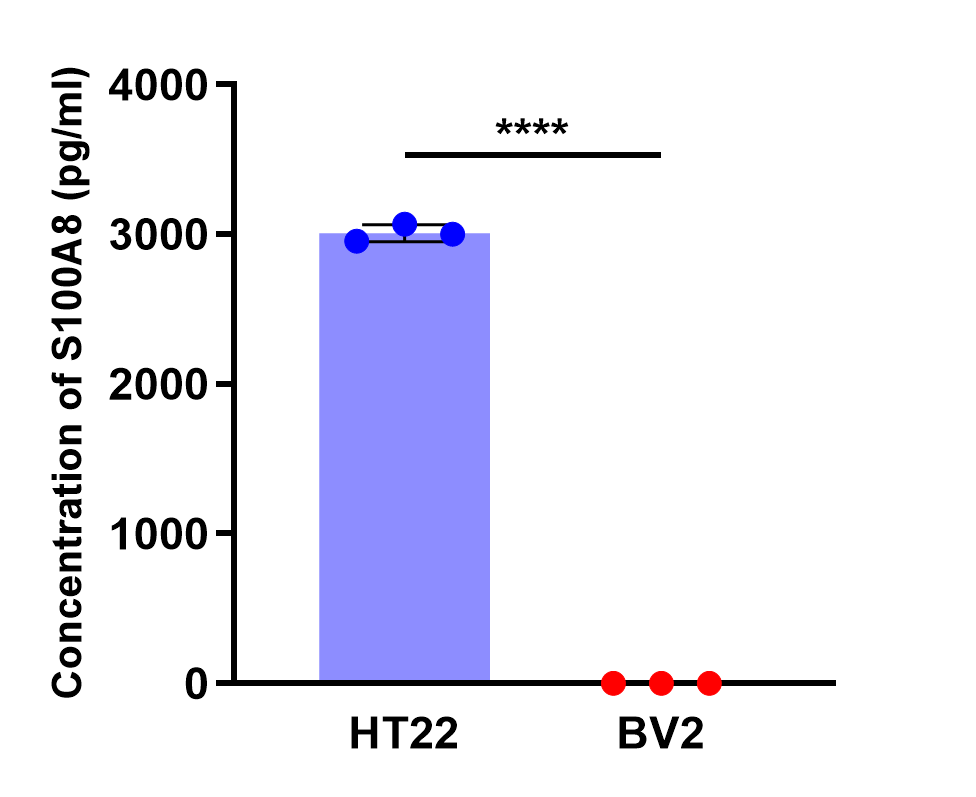


Figure S10. ELISA analysis of S100A8 protein concentration in the culture supernatants of HT22 cells and BV2 cells. Data are presented as mean ± SD from n = 3 independent experiments. Statistical significance was analyzed using one-way ANOVA followed by Tukey’s multiple-comparison test. ****p < 0.0001.


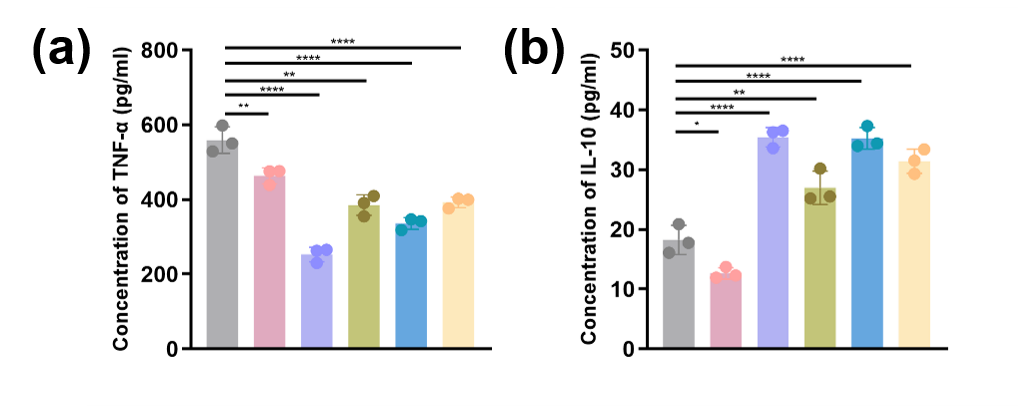


Figure S11. ELISA quantification of the TNF-α (a) and IL-10 (b) in the co-culture supernatant. Data are presented as mean ± SD from n = 3 independent experiments. Statistical significance was analyzed using one-way ANOVA followed by Tukey’s multiple-comparison test. *p < 0.05; **p < 0.01; ***p < 0.001; ****p < 0.0001.


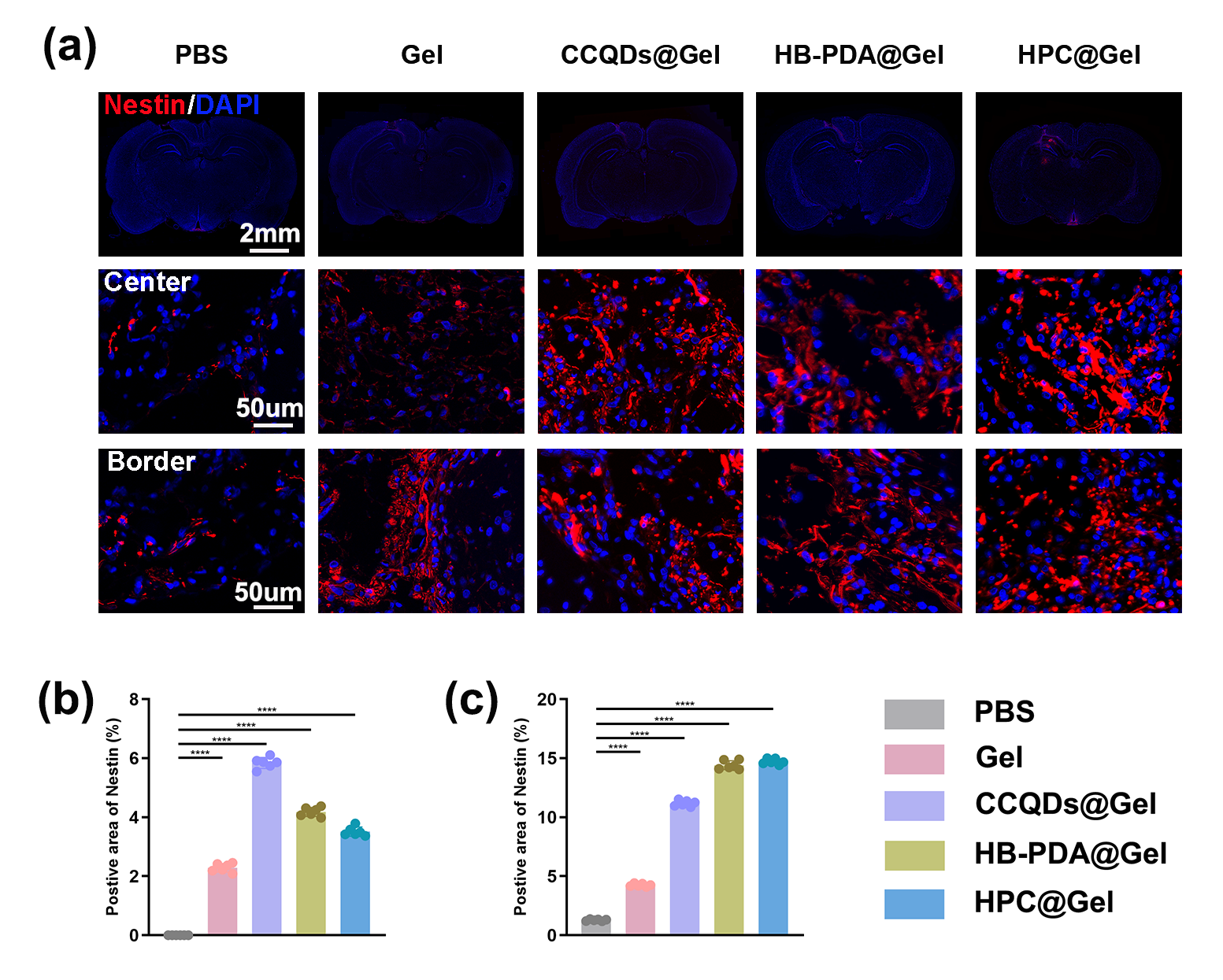


Figure S12. (a) Representative immunofluorescence images of the neural progenitor cell marker Nestin (red) in the lesion center and border at 28 days post-TBI. (b, c) Quantitative analysis of the Nestin-positive area in the lesion (b) center and (c) border. Data are shown as mean ± SD (n = 6 animals per group). Group differences were evaluated using one-way ANOVA, followed by Tukey’s multiple-comparison test. Significance levels are indicated as *p < 0.05, **p < 0.01, ***p < 0.001, ****p < 0.0001.


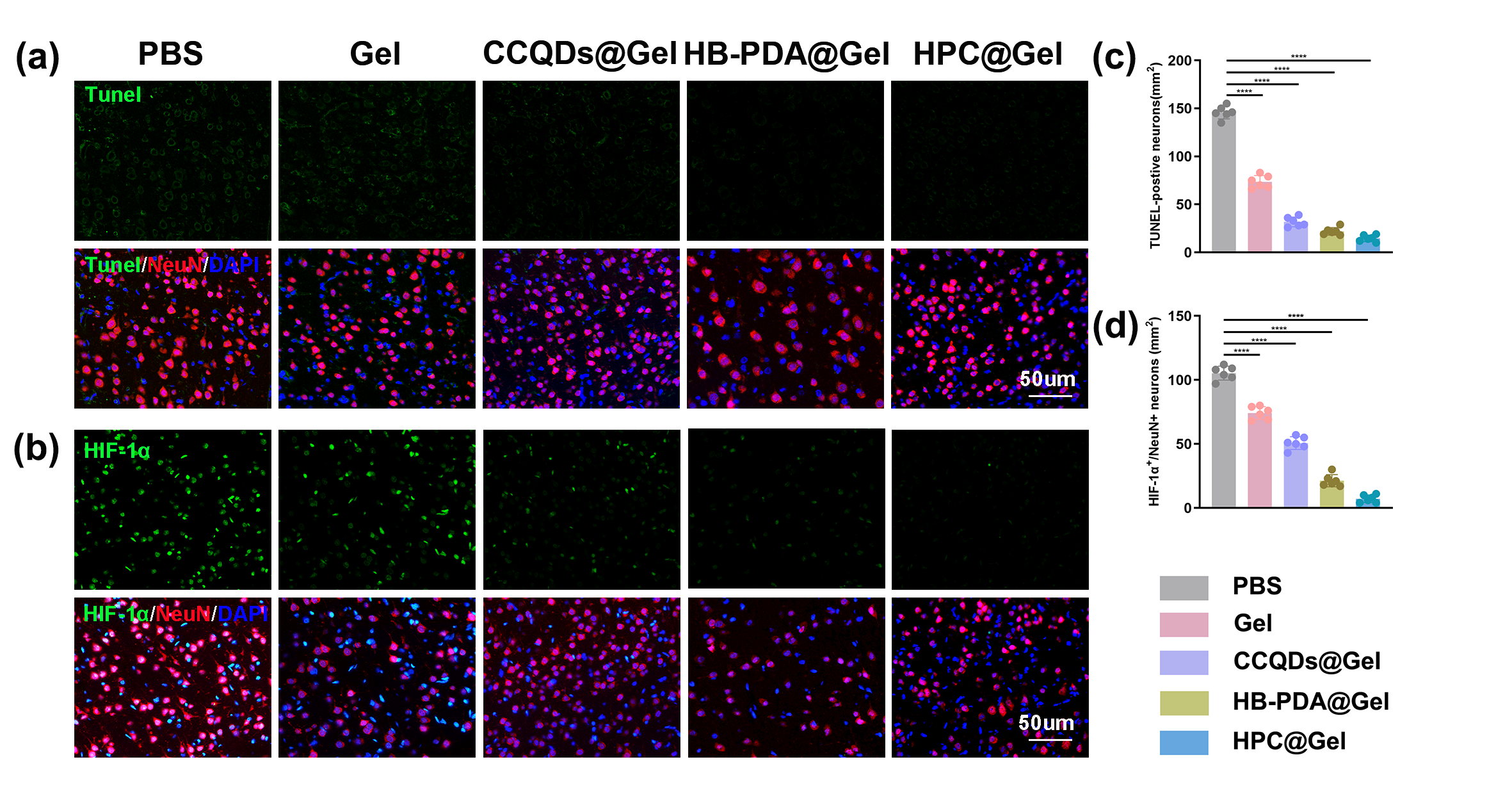


Figure S13. The biomimetic hydrogel system confers in vivo neuroprotection at 7 days post-TBI. (a) Representative immunofluorescence images showing apoptotic cells (TUNEL, green) and neurons (NeuN, red) in the pericontusional cortex. (b) Representative immunofluorescence images showing hypoxic cells (HIF-1α, green) and neurons (NeuN, red). (c) Quantification of TUNEL-positive neurons. (d) Quantification of HIF-1α-positive neurons. Data are presented as mean ± SD (n = 6 animals per group). Statistical significance was analyzed using one-way ANOVA followed by Tukey’s multiple-comparison test. *p < 0.05, **p < 0.01, ***p < 0.001, ****p < 0.0001.


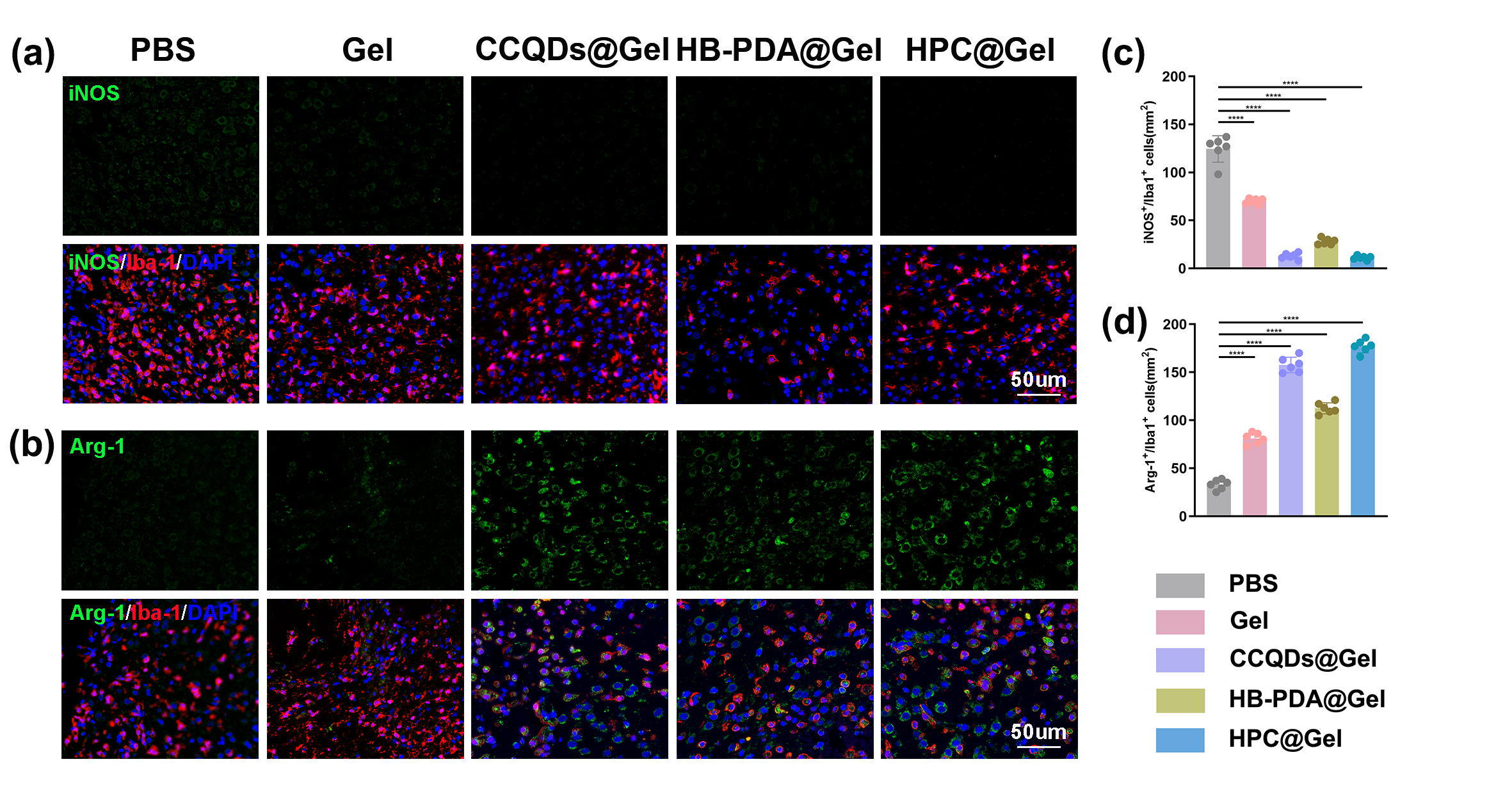


Figure S14. (a) Representative immunofluorescence images showing iNOS (green) and Iba-1 (red). (b) Representative immunofluorescence images showing Arg-1(green) and Iba-1 (red). (c) Quantification of iNOS-positive microglia cells. (d) Quantification of Arg-1-positive microglia cells. Data are presented as mean ± SD (n = 6 animals per group). Statistical significance was analyzed using one-way ANOVA followed by Tukey’s multiple-comparison test. *p < 0.05, **p < 0.01, ***p < 0.001, ****p < 0.0001.


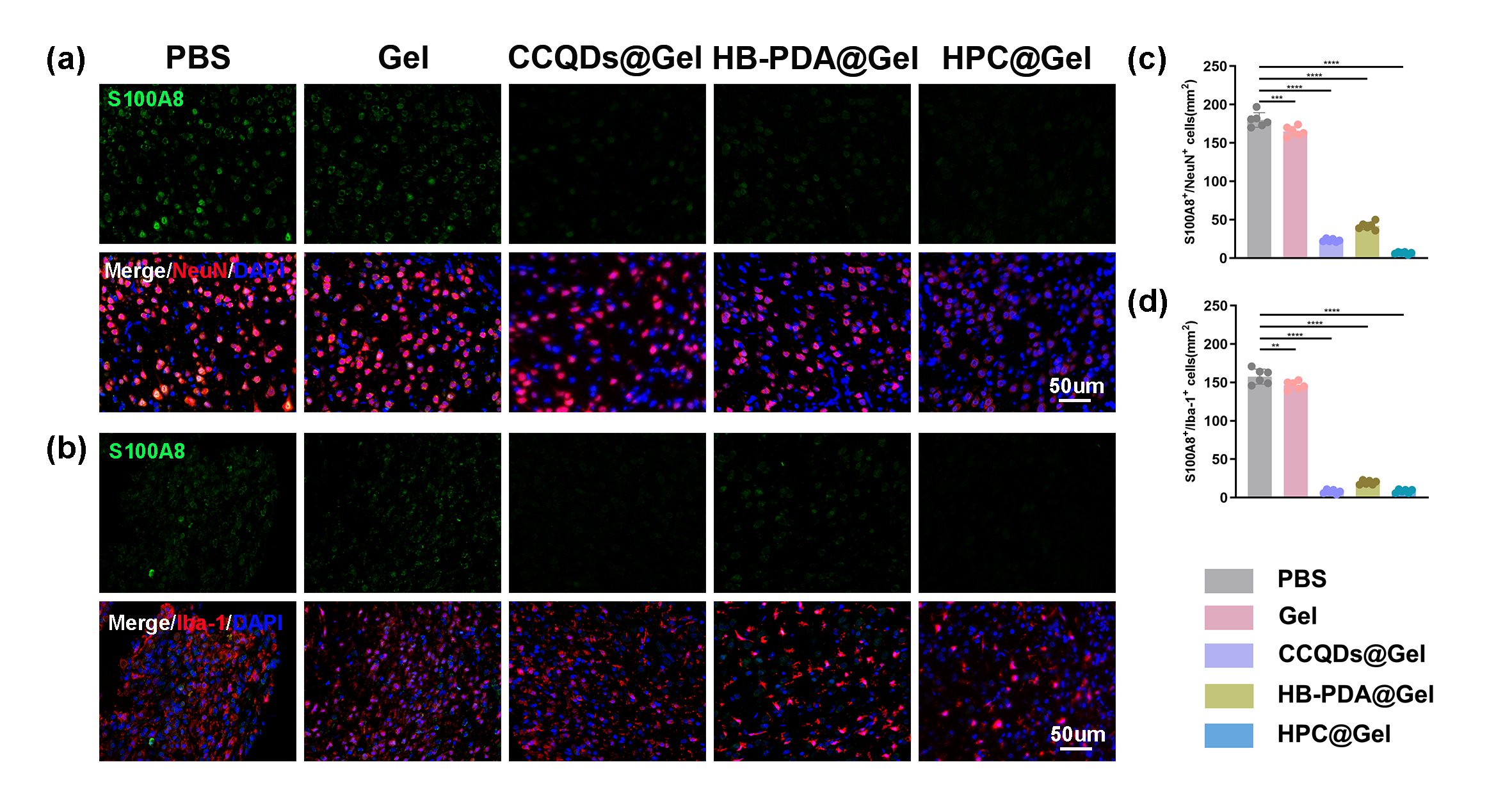


Figure S15. (a, b) Representative immunofluorescence images of S100A8 (green) co-stained with (a) NeuN (red) and (b) Iba-1 (red) in the TBI lesion area. Nuclei are counterstained with DAPI (blue). (c, d) Quantitative analysis of the number of (c) S100A8+/NeuN+ cells and (d) S100A8+/Iba-1+ cells. Data are presented as mean ± SD (n = 6 animals per group). Statistical significance was analyzed using one-way ANOVA followed by Tukey’s multiple-comparison test. *p < 0.05, **p < 0.01, ***p < 0.001, ****p < 0.0001.

Table S1. RNA-seq data quality metrics.

| Sample | Clean reads | Error rate(%) | Q20(%) | Q30(%) | GC content(%) | Unique mapped |
| --- | --- | --- | --- | --- | --- | --- |
| PBS_1 | 39545178 | 0.012 | 99.13 | 95.79 | 48.76 | 37673698(95.27%) |
| PBS_2 | 46093692 | 0.0121 | 99.11 | 95.76 | 50.18 | 44010662(95.48%) |
| PBS_3 | 44031846 | 0.0123 | 99.03 | 95.35 | 51.16 | 42025477(95.44%) |
| HPC@Gel_1 | 47905168 | 0.0122 | 99.05 | 95.5 | 51.28 | 45653250(95.3%) |
| HPC@Gel_2 | 43922476 | 0.0123 | 99.01 | 95.24 | 51.68 | 41858503(95.3%) |
| HPC@Gel_3 | 43148214 | 0.0122 | 99.03 | 95.42 | 52.24 | 41052083(95.14%) |


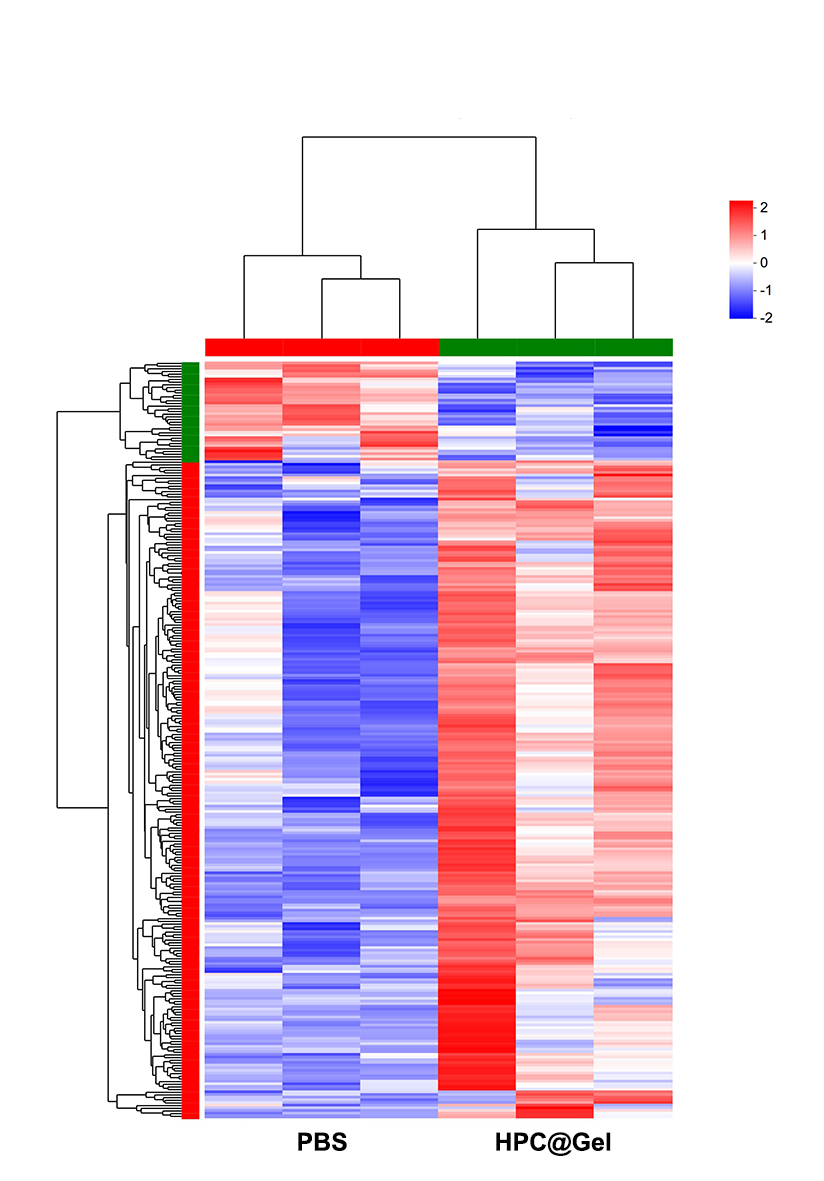


Figure S16. Heatmap showing expression patterns of DEGs (padj < 0.05, |log2FC| > 1) in PBS and HPC@Gel samples. Rows are genes, columns are samples. Normalized expression values were row-scaled (Z-score). Red: high expression; blue: low expression. The dendrograms represent hierarchical clustering (Euclidean distance, complete linkage).


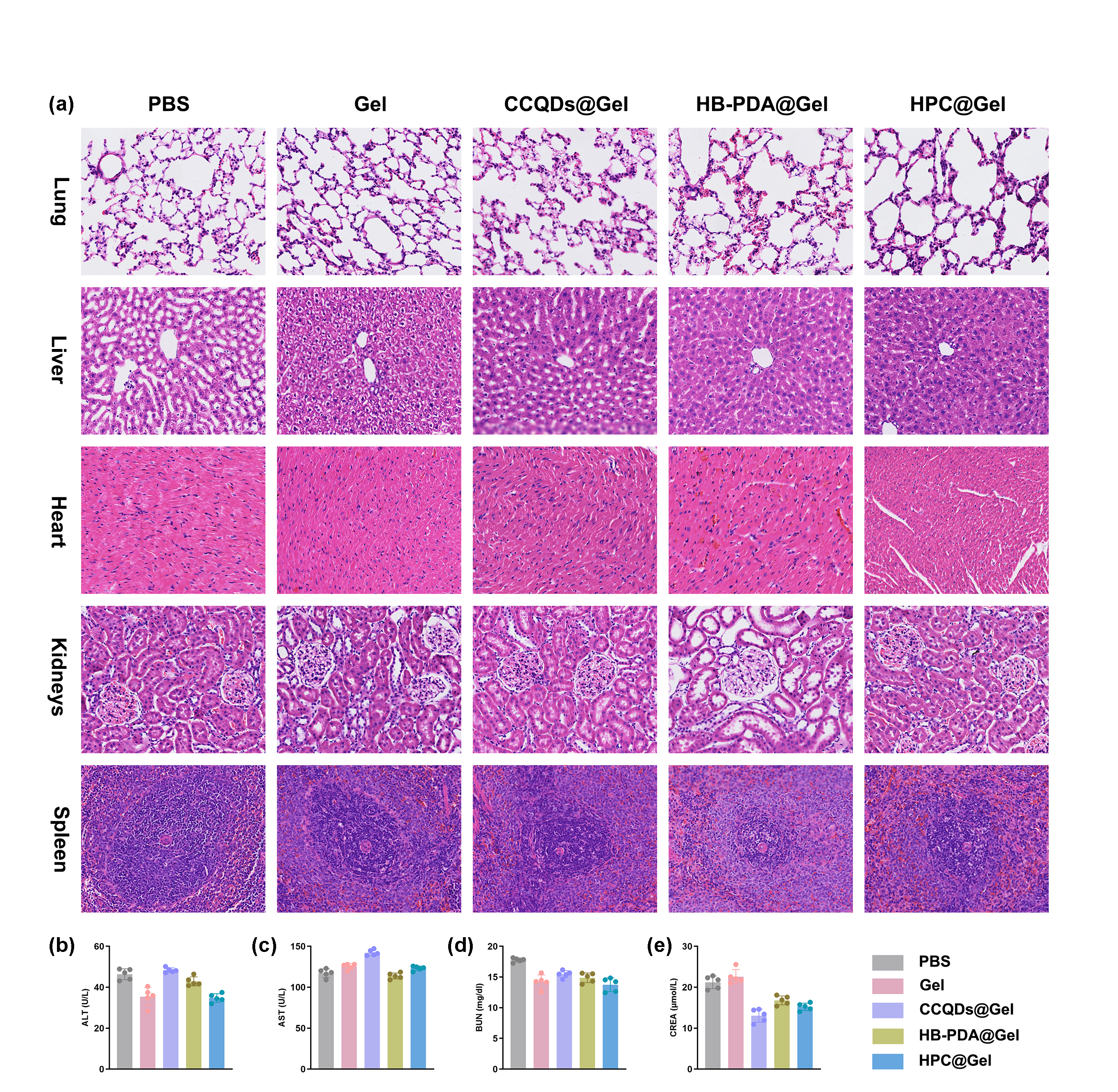


Figure S17. (a) Representative HE-stained sections of the heart, liver, spleen, lung, and kidney at 28 days post-TBI. Serum levels of (b) ALT, (c) AST, (d) BUN, and (e) CREA measured from venous blood. Data are presented as mean ± SD (n = 6 animals per group).
